# Second primary cancers and hormonal therapies for prostate cancer: A nested case–control study

**DOI:** 10.1111/fcp.70004

**Published:** 2025-03-17

**Authors:** Lucie‐Marie Scailteux, Julien Bezin, Marion Gundelwein, Julien Edeline, Emmanuel Oger, Frédéric Balusson, Antoine Pariente

**Affiliations:** ^1^ Univ Rennes, Inserm, EHESP, Irset (Institut de recherche en santé, environnement et travail) ‐ UMR_S 1085 Rennes France; ^2^ Pharmacovigilance, Pharmacoepidemiology and Drug Information Centre Rennes University Hospital Rennes France; ^3^ Service de Pharmacologie Médicale, CHU Bordeaux ; Bordeaux Population Health Research Center, Team Pharmacoepidemiology, UMR 1219, Inserm, DRUGS‐SAFE National Platform of Pharmacoepidemiology University of Bordeaux Bordeaux France; ^4^ Department of Medical Oncology, CLCC Eugène Marquis, COSS (Chemistry Oncogenesis Signaling) INSERM, University Rennes Rennes France

**Keywords:** abiraterone, enzalutamide, safety, second primary cancer

## Abstract

**Introduction:**

(Pre‐)clinical studies have not ruled out a potential risk of second primary cancer (SCP) under the effect of some new androgen receptor pathway inhibitors (ARPIs), especially enzalutamide (ENZ).

**Methods:**

Using the French health reimbursement claims database (Système National des Données de Santé), we designed a case–control study nested in a 2013–2020 cohort of new users of androgen‐deprivation therapy. The cases were patients with a first diagnosis of SPC, identified beyond 12 months following cohort entry and up to December 31st^,^ 2021; up to 10 controls were matched per case, based on age and cohort entry date. The main analysis focused on patients who had not switched to a different ARPI. Applying a one‐year lag time, we determined the most frequent and longest cumulative exposure patterns to abiraterone (ABI) or ENZ and estimated the odds ratios.

**Results:**

The cohort comprised 147 092 patients, including 7928 cases and 78 554 controls eligible for analysis. The SPCs mainly involve the digestive organs, the urinary tract, or the lungs. Recent and short exposure to ENZ was associated with SPC: OR 1.7, 95% CI [1.2–2.4]. Recent one full year of exposure to ABI, as well as full‐year plus part of the second year, was associated with SPC: OR 1.8 [1.2–2.7] and 2.3 [1.3–4.0], respectively.

**Discussion/Conclusion:**

SPC cases were mainly observed among recently exposed patients, which could be linked to a detection bias. The insufficient number of patients exposed over many years means that no definitive conclusions can be drawn.

**List of abbreviations**
ABI: abiraterone
ADT: androgen‐deprivation therapy
ARPIs: androgen receptor pathway inhibitors
ATC: Anatomical Therapeutic Chemical
ENZ: enzalutamide
ICD‐10: International Classification of Diseases, 10th edition
OR: odds ratio
PSA: stands for prostatic specific antigen
PY: person‐years
SNDS: Système national des données de santé
SPC: second primary cancer
US: United States
WCE: weighted cumulative exposure
95%IC: 95% interval confidence

## 1 INTRODUCTION

Used in association with androgen‐deprivation therapy (ADT), a number of new androgen receptor pathway inhibitors (ARPIs; abiraterone [ABI]/Zytiga® (Janssen‐Cilag International NV, Beerse, Belgium), apalutamide/Erleada® (Janssen‐Cilag International NV, Beerse, Belgium), darolutamide/Nubeqa® (Bayer AG, Leverkusen, Germany), and enzalutamide [ENZ]/Xtandi® (Astellas Pharma Europe B.V., Leiden, Netherlands)) are well‐recognized prostate cancer treatments, initially used mainly in castration‐resistant prostate cancer [1]. While ABI and ENZ have been used since the early 2010s, apalutamide and darolutamide have only been used since 2020.

Issues have arisen in marketing authorization applications following approval for the metastatic castration‐resistant stage, when non‐clinical data pointed to the potential carcinogenicity of ENZ, with tumors observed in the thymus, the pituitary gland, and the bladder [2]. Although no mechanism has been established, urothelial carcinoma is thought to be induced by permanent local epithelium irritation by ENZ calculi and crystals observed in rat bladders [2, 3]. In comparisons with placebo, cases of secondary primary cancer (SPC) (bladder/urothelium, colon) have been observed in quite similar or higher proportions in ENZ phase‐3 trials [4, 5, 6], which justified a special warning in the ENZ/Xtandi® summary of product characteristics [7]. Patients are advised to consult their doctor if they notice signs suggestive of urinary or digestive malignancies. With ABI and darolutamide, animal data does not suggest a carcinogenic effect [8, 9]. Although testicular neoplasms have been observed in rats treated with ABI, the data indicates that this species is genetically predisposed to this type of tumor [8, 10, 11, 12]. Apalutamide animal data has reported some malignancies (testicular, mammary, thyroid, etc.) but they were also considered as rat‐specific [13]. No malignant‐type adverse events have been identified in the ABI, apalutamide, and darolutamide phase‐3 trials [14, 15, 16, 17, 18, 19, 20]. Unlike the darolutamide/Nubeqa® risk management plan, which mentions potential carcinogenicity among important potential risks [21], malignancies are not mentioned as a safety concern with ABI, ENZ, and apalutamide [2, 8, 22].

To date, no study has assessed the risk of developing SPCs among ARPI users. Studies have focused on describing SPC and/or on estimating the incidence among prostate cancer survivors [23, 24], and sometimes more specifically among castration‐resistant prostate cancer cohorts in Germany, Sweden, and the United States [25]. Overall, the study periods included patients at a time when ABI and ENZ were in clinical development or were starting to be used, and no treatment‐related risk assessment was performed. Now that the marketing of ARPIs for earlier stages of prostate cancer [26] is being considered, more knowledge is needed, because the concomitant risk, if there is one, of developing SPC as a result of using one of the ARPIs could alter patients' overall survival.

The aim of the study was thus to assess the impact of ARPI exposure on subsequent risks of developing SPCs in a prostate cancer population.

## 2 MATERIALS AND METHODS

### 2.1 Study design, setting and patients

We performed a nested case–control study using the *Système National des Données de Santé* (SNDS) as the data source for the 2009–2021 period. The study was not registered. The protocol was submitted to the GIS EPI‐PHARE so the study could be included in the research program of the Drugs‐Safe®‐GIS EPI‐PHARE partnership funded by the French Medicines Agency ANSM. The protocol is available as a supplementary material. We followed the RECORD‐PE reporting guideline and the checklist is provided in supplementary material.

The SNDS collates pseudonymized medical‐administrative data, covering 99% of the French population, providing information on patients (age, gender, vital status, etc.). It contains information on all healthcare acts that lead to reimbursement (dispensing medication, biological and radiological acts, consultation, hospitalization) by the health insurance system (*Assurance Maladie)*. Healthcare reimbursements are identified through classifications such as the Anatomical Therapeutic Chemical (ATC) classification for drugs, medical procedure codes, or the International Classification of Diseases, 10th edition (ICD‐10) for disease diagnoses [27].

The cohort entry selection criteria were: a first reimbursement for ADT in the period 2013 to 2020 (the cohort entry date was the ADT initiation date), without ADT or ABI or ENZ reimbursement identified from 2009 to 2012; age ≥ 40 years; a diagnosis of prostate cancer around the date of entry into the cohort (i.e. a prostate cancer diagnosis using the C61 or D400 ICD‐10 codes before or in the 3 months after initiation, or two prescriptions for dosing the prostatic specific antigen or one prostate biopsy in the 12 months before ADT initiation); and without a history of cancer other than prostate cancer before the cohort entry date (see Table S1 for details).

We identified cases as patients with an SPC diagnosis (ICD‐10, C00‐C96; Table S1) after the ADT initiation date (with the exception of prostate cancer, and non‐melanoma skin cancers) up to December 31st, 2021. Not all cases of SPC were of interest for the analysis, given a potential detection bias for patients for whom an ICD‐10 diagnosis code C77‐C80 (metastatic cancer codes) or D00‐D48 (benign neoplasm or of unspecific) were first observed. As these patients may be monitored more closely, the detection of a SPC is more likely than for patients without these codes (see Figure S1). In an attempt to exclude all active cancers other than prostate cancer, we also excluded cancers diagnosed in the first year following the initiation of ADT (Figure S1). Up to 10 controls were matched to the cases using an incidence density sampling design, based on the same month and year of birth, and the same cohort entry date (± 3 months to facilitate matching of controls to cases), with replacement.

### 2.2 Exposure

The exposure of interest was ARPI (Table S1), especially ABI and ENZ, for which initiation and subsequent use were identified from monthly reimbursements. Apalutamide and darolutamide use was expected to be infrequent given the study period and their recent market authorization in France. It is difficult to estimate the impact of a switch between ARPIs on the risk of SPC, and above all, to distinguish which ARPI drug could alter the SPC risk, and in what way. Cases (and their related controls), for whom exposure included ARPI switches, were thus not considered for the case–control analysis. Several exposure groups were considered: ARPI non‐users (defined as being not exposed to ARPIs), ABI new users, and ENZ new users, regardless of ADT use for these two groups (see Figure S2). Before the SPC diagnosis date (and the related date for controls), ABI and ENZ cumulative exposure took the date of each reimbursement and the dispensing of one month's treatment into account; the dispensing date was used as the treatment starting date. An annual ratio of cumulative exposure ratio was estimated by dividing the number of supposed daily‐exposed patients by 365. Daily exposure to either ABI or ENZ was deduced from the quantity delivered (number of pills) as recorded in the healthcare reimbursement database. To facilitate the representation of cumulative exposure, we considered as “unexposed”, “partially‐exposed” and “fully exposed”, patients exposed < 25%, ≤ 25% and < 75% and ≥ 75% per year to ABI or ENZ, respectively. In some cases, given that it takes a number of years to observe clinical manifestations and diagnose a cancer linked to a causal agent [28, 29], i.e. cancer latency, we applied a one‐year lag time, not considering ARPI exposure in the year immediately preceding the SPC diagnosis date (and the related date for the controls).

### 2.3 Statistical analysis

**FIGURE 1:**
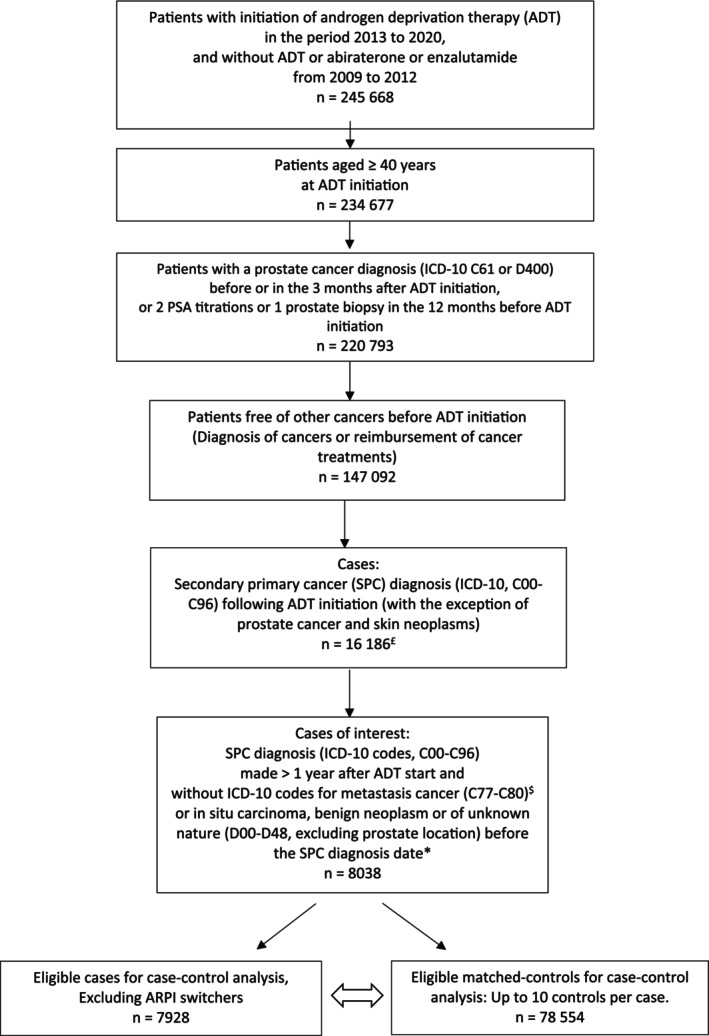
Flowchart. ADT: androgen deprivation therapy; ARPIs: androgen receptor pathway inhibitors. ^$^ Because metastatic cancer codes (C77‐C80) potentially have low sensitivity and/or specificity, and metastatic prostate cancer progression could be coded as a second primary cancer, a potential classification bias could exist. We therefore decided to exclude the metastatic cancer codes. * Cases with ICD‐10 codes C77‐C80 or D00‐D48 (benign neoplasm or of unknown nature) identified before C00‐C96 were excluded because of a potential detection bias. This is because patients with C77‐C80 or D00‐D48 first could receive closer monitoring and could be more likely to be diagnosed with a second primary cancer than patients without these codes first. See appendix Figure S1.

First of all, we estimated the incidence of SPCs in the cohort, taking all cases of interest into account (Figure 1).

Regarding case–control analyses on ARPI non‐switchers, patient characteristics (comorbidities included in the Charlson score identified in the 3 years before inclusion; radiotherapy and radical prostatectomy, retrospective up to 01/01/2009), were described at the time of cohort entry according to their case and control status, on an individual basis (as some of the controls could be used several times, and some cases could be controls for other cases).

The SPC location was described, as well as cancers in a pelvic location (rectal, bladder cancer, etc.) that could be related to prostate cancer radiotherapy [1, 30, 31, 32, 33].

We ran conditional logistic regression models to estimate the odds ratios (95% CI) for cumulative exposure to ABI or ENZ (< 1 year, 1–2 years, 2–3 years, > 3 years), using the ARPI non‐user group as the reference, and a one‐year lag time to address cancer latency.

We also described the patterns of exposure to ABI or ENZ, in particular the most frequent (with at least 20 patients per pattern), and, if different, those for which the duration of exposure was the longest. A one‐year lag time was also applied. A similar exploration was made for the most frequent SPC locations distinguishing digestive and urinary cancers. We then ran conditional logistic regression models to estimate the odds ratios (95% CI) for each pattern of ABI or ENZ exposure (applied with a one‐year lag time), using the ARPI non‐user group as the reference.

As an exploratory approach, the weighted cumulative exposure (WCE) method was used to assess the impact of the period of ARPI exposure in addition to its intensity and duration over a prespecified time period [34, 35]. This exploratory approach was not planned in the initial protocol provided in the supplementary material. Estimated from data observed in the study, i.e. as data‐driven model, it considers the cumulative weighted sum of the effect of the past exposures, to estimate with flexible cubic splines the weight function of the relative importance of past doses on the current risk [36, 37, 38].

## 3 RESULTS

The source cohort included 147 092 patients who started an ADT treatment in the period from 2013 to 2020. Among these, 8038 cases of SPC were identified up to the end of 2021 (Figure 1), corresponding to an incidence of 1.33 per 100 person‐years (PY) (95% CI, 1.31–1.36).

Focusing on ARPI non‐switchers, 7928 cases and 78 554 controls (corresponding to 48 296 individuals used for controls) were considered for the main analysis (Figure 1). The baseline characteristics are shown in Table 1. The mean age was 75 years for both cases and matched controls. A history of radiotherapy for prostate cancer was identified for 4%. The main SPCs identified among the cases involved digestive organs (about 35%), the urinary tract (22%), and the lungs (about 13%); cancers in areas potentially exposed to prostate cancer radiotherapy accounted for 24% of the SPCs (the bladder and genitourinary tract, 17.4%, and the colorectal gastrointestinal tract, 6.8%, respectively). The types of SPC are detailed in Table S2. The conditional odds ratios for SPCs stratified on cumulative drug exposure are shown in Table S3.

**TABLE 1:** Baseline patient characteristics among the eligible cases and matched controls, based on individuals.

	Eligible cases (n = 7928)	Matched controls (n = 48 296)	Standardized difference
Mean age (SD), year	75.3 (8.1)	75.0 (8.4)	0.0420
Previous prostate cancer management, n (%)			
Prostatectomy	775 (9.8)	5664 (11.7)	−0.0631
Radiotherapy	345 (4.4)	2093 (4.3)	0.0009
Comorbidities, n (%)			
Myocardial infarction	219 (2.8)	1321 (2.7)	0.0017
Ischemic heart disease	1188 (15.0)	6578 (13.6)	0.0390
Congestive heart failure	363 (4.6)	1914 (4.0)	0.0305
Peripheral vascular disease	775 (9.8)	3006 (6.2)	0.1312
Cerebrovascular disease	454 (5.7)	2718 (5.6)	0.0043
Dementia	199 (2.5)	1371 (2.8)	−0.0204
Chronic pulmonary disease	1481 (18.7)	7632 (15.8)	0.0762
Rheumatologic disease Connective tissue disease	84 (1.1)	521 (1.1)	−0.0019
Peptic ulcer disease Ulcer disease	47 (0.59)	222 (0.5)	0.0184
Mild liver disease	127 (1.6)	420 (0.9)	0.0663
Moderate or severe liver disease	21 (0.3)	64 (0.1)	0.0297
Diabetes without complication	1482 (18.7)	8123 (16.8)	0.0491
Diabetes with end‐organ damage	277 (3.5)	1225 (2.5)	0.0560
Hemiplegia	116 (1.5)	658 (1.4)	0.0085
Moderate or severe renal disease	265 (3.3)	1451 (3.0)	0.0193
AIDS/HIV	25 (0.3)	91 (0.2)	0.0253

SD: standard deviation; AIDS/HIV: Acquired Immune Deficiency Syndrome/human immunodeficiency viruses‐1.

**FIGURE 2:**
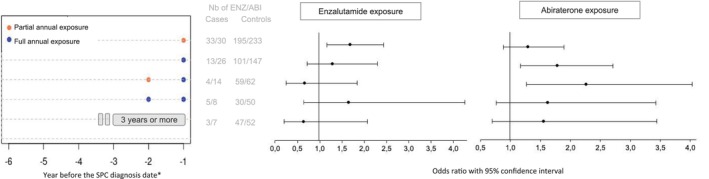
Patterns of abiraterone or enzalutamide exposure and related conditional odds ratios. ABI: abiraterone; ENZ: enzalutamide; SPC: second primary cancer. *A one‐year lag time was applied, not considering the year of drug exposure before the SPC initiation date.

After applying a one‐year lag time, the most frequently identified patterns of exposure concerned exposure > 1 year to ARPIs before the SPC diagnosis, for patients partially (ENZ: n = 228, ABI: n = 263) or fully exposed (ENZ: n = 114, ABI: n = 173) (Figure S3; patterns evaluated without the lag time: Figure S4‐S5). In contrast with ENZ, an association of ABI use with the risk of SPC was found to be significant for one full year of exposure and for one full year plus part of the second year of exposure to ABI (OR: 1.8, 97.5% CI [1.2–2.7] and 2.3 [1.3–4.0], respectively) (Figure 2; Figure S3). Cumulative drug exposure > 3 years concerned few patients, thus preventing us from distinguishing between full and partial exposure; associations with the risk of SPC were found non‐significant although in opposite directions for ABI and ENZ (OR: 1.5, 97.5% CI [0.7–3.4], and 0.6 [0.2–2.1], respectively) (Figure 2).

**FIGURE 3:**
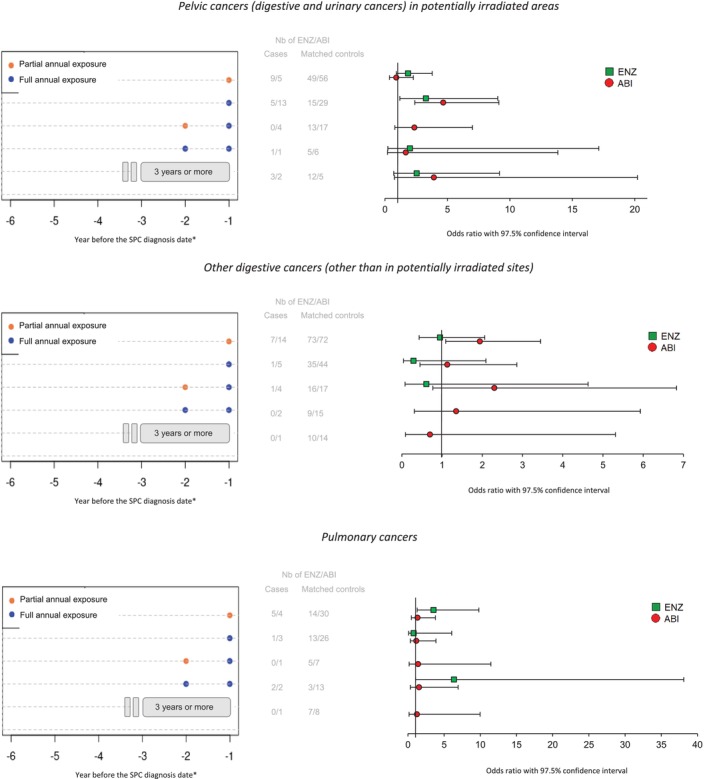
Patterns of abiraterone or enzalutamide exposure and related conditional odds ratios stratified by type of SPC. ABI: abiraterone; ENZ: enzalutamide; SPC: second primary cancer. * The patterns shown involve a one‐year lag time, not considering drug exposure in the year before the cancer diagnosis.

The results are detailed by cancer type in (Figure 3). Regardless of the pattern of exposure, the risk of SPC occurrence increased for ABI, but not for ENZ for digestive cancers and not for either of these ARPI drugs for pelvic cancers. Analyses were inconclusive for pulmonary cancers, for which the number of cases was very small. The results of the sensitivity analyses using the WCE method were non‐significant for both ABI and ENZ exposure Figure S6.

## 4 DISCUSSION

Using a 2013–2021 nested case–control study, we estimated SPC incidence to be 1.33 per 100 PY in a French nationwide cohort of prostate cancer patients, who were new users of ADT. This incidence was lower than that estimated in three castration‐resistant prostate cancer cohorts in the US, Germany, and Sweden (5.9 (95% CI: 5.0–6.8), 7.9 (95% CI: 7.0–8.8), 10.2 (95% CI: 9.0–11.5) per 100 PY, respectively) [25, 39, 40]. The difference could relate to the fact that we did not consider all cancers for the analysis. For instance, as in the US study, we did not consider non‐melanoma skin cancers in our definition. Another source of difference could relate to the outcome identification procedure: using the SNDS database, SPC diagnoses relied only on in‐hospital databases, probably at a later stage of the disease (early diagnoses related to outpatient visits or/and physician files were not available); an increase in the number of patient‐years could have decreased the incidence rate. Finally, we could have missed early deaths from aggressive cancers among patients who did not come to the hospital. Overall, cancer locations were similar across studies including ours (urinary/bladder, colon/non‐colorectal, lung), with somewhat different distributions [25]. The SPC distribution identified in our study is consistent with that observed in prostate cancer survivors [23, 24]. The interpretation of our results on localized cancers remains difficult, particularly for cancers in areas potentially irradiated for prostate cancer, as our data source and study period did not enable us to confirm the use of radiotherapy for all patients.

Considering ARPI exposure more in‐depth, in our analysis, few patients had ARPIs switches, allowing most of our cases of interest to be retained in the case–control analysis. This is consistent with previous data indicating that a majority of patients reaching castration‐resistant stage receive only one line of ARPI during follow‐up or before death [41]. Regardless of how exposure is taken into account, from overall cumulative exposure or from annual patterns, our results suggest that SPCs were mostly observed among patients with a relatively short exposure (1 to 2 years, excluding the year before the SPC initiation date). This could be compatible with a global detection bias, as patients who had recently progressed to the castration‐resistant stage probably had more frequent contact with healthcare professionals. Close monitoring of patients for whom cancer has progressed and who are using a new treatment is recommended in order to detect early treatment failure and/or an adverse drug reaction [1]. In addition, ABI and ENZ are known to induce adverse drug reactions in the months following their introduction, including serious reactions requiring medical care [42, 43]. However, we cannot exclude a differential bias due to the fact that the management of patients under ENZ or ABI differs. In particular, ABI is to be used in combination with corticosteroids to suppress the action of the adrenocorticotropic hormone (ACTH) and to limit the occurrence and severity of hypertension, hypokalemia, fluid retention, and cardiac failure [44].

To determine whether there is a truly significant association between short, recent exposure to ABI or ENZ and SPC occurrence, a pathophysiological rationale should be considered. Despite the fact that the cancer latency time is not always known and can vary across cancer types, authors have agreed on a period of several years, and even decades, after carcinogen exposure [28, 45], which contrasts with our results. In addition, if a causal relationship existed, we hypothesize that ARPIs, especially ENZ, could be carcinogenic promoters, i.e. drugs that accelerate the progression or growth of subclinical/premalignant disease [28]. An initiating process, inducing a first clone of neoplastic cells, mostly through genotoxic effects (e.g. phenacetin and upper urinary tract cancer [46]; radioactive iodine and leukemia or solid cancers [47]), and supposing longer carcinogenic exposure [28], is not plausible.

To take cancer latency and screening bias into account, we used one‐year lag time periods (firstly ignoring SPC occurring within the first year of the cohort entry, and secondly, not taking the last year of exposure before the SPC initiation date into account). Using longer lag times, as suggested by other authors [28] could have been a possibility, to the detriment of the follow‐up duration. However, this would have mainly had an interest if an association had been found, in order to strengthen the controlling for reverse causality bias. Furthermore, we ran different statistical models to enable several hypotheses regarding hazard functions to be taken into account. We first used logistic regression models which considered cumulative ARPI exposure. This entails a strong a priori assumption, that the impact of past exposure on hazard functions is the same regardless of when the patient was exposed and for how long. As this could be highly questionable [35], we analyzed the patterns of ARPI exposure (like others [48]) which also provided a good description of ARPI use from 2013 to 2021. Finally, we tried the weighted cumulative exposure method, which was specifically developed to take the intensity, duration, and period of drug exposure [34] into account. However, at the time of analysis in our study, the R package was not fully developed for case–controls and nested case–control designs [38], and we had little exposure data to input into the model.

An evaluation of long‐term associations was inconclusive given that in our study, fewer than 60 patients had 3 years or more exposure to ABI or ENZ, excluding the year before the SPC initiation date. In addition, long‐term exposure implies fairly long survival, but at the metastatic castration‐resistant prostate cancer stage, median overall survival is around 2 to 3 years [49, 50, 51], and about 10% of ABI/ENZ new users died early in the first year [41]. We need to give clinicians time to use these treatments at earlier stages of prostate cancer, and then monitor SPC occurrence so as assess the long‐term associations more efficiently.

Among the limitations of this work, we did not take all specific cancer risk factors, such as smoking, alcohol consumption, or environmental exposure, into account, as they are poorly or not at all identified in our data source. Residual confounding (especially with smoking) could not be ruled out as well as a detection bias; thus, no firm conclusion can be drawn. However, we assume that comorbidities or risk factors are not confounders because they do not influence drug choice (ABI vs ENZ). The date of SPC identified in the SNDS is potentially later than in reality. But this misclassification bias is thought to be non‐differential under the assumption that medical follow‐up is more or less comparable. Of note, the number of medical doctor (including specialist) consultations as well as the reimbursement of cancer screening measures (including biological and imaging) were well balanced (data not shown). Over the study period, we could not assess the risks of apalutamide or darolutamide exposure as they had been marketed too recently.

One of our strengths was the use of a national claims database, which enabled us to identify a rare event, SPC, and to carry out analyses by drug exposure; but we acknowledge potentially insufficient information on ARPI exposure and SPCs to draw any definitive conclusions.

## 5 CONCLUSION

In a cohort of new ADT users, we estimated that SPC was a very uncommon event, and this is good news. The few cases of SPC observed were among patients recently exposed to ABI or ENZ and for less than 3 years: this was unexpected and could be the result of chance, or detection bias. Unfortunately, long‐term exposure to ABI or ENZ could not be adequately assessed, nor exposure to apalutamide or darolutamide. In the meantime, we might conclude that this study has not identified any alarming signals that would warrant reconsideration of usual care, or of usual awareness.

## AUTHOR CONTRIBUTIONS

Scailteux and Balusson had full access to all of the data that was used to generate the study population. The database extracted was stored in a dedicated space on the CNAMTS portal. M. Balusson carried out the data management (cleaning, table design, and choice of variables for statistical analyses). They take responsibility for the integrity of the data and the accuracy of the data analysis.

Conception and design: Scailteux, Oger.

Acquisition, analysis, and interpretation of the data: All authors.

Drafting of the manuscript: Scailteux.

Critical revision of the manuscript for important intellectual content: All authors.

Statistical analysis: Balusson, Gundelwein, Scailteux, Oger.

Obtained funding: Scailteux, Pariente.

Administrative, technical, or material support: Pariente.

Supervision: Oger, Pariente.

Critique of completed data analysis and interpretation in the manuscript: all authors.

## CONFLICT OF INTEREST STATEMENT

The authors declare no conflicts of interest.

## ETHICS STATEMENT

This work was supported by the French Drugs Agency as part of the DRUG‐SAFE® research program. According to the provisions of the French Data Protection Supervisory Authority (Commission Nationale de l'Informatique et des Libertés), neither approval from the ethics committee nor informed consent was required for this observational study based on the French anonymized medical‐administrative database.

## Supporting information


**Table S1.** Details of codes.
**Table S2.** Details of the second primary cancer codes (n = 8038) by decreasing frequency.
**Table S3.** Conditional odds ratios for second primary cancer stratified by drug cumulative exposure.
**Figure S1.** Case–control eligibility. A – for cases. B – for controls.
**Figure S2.** Drug exposure group among eligible cases (in the population not switching ARPI).
**Figure S3.** Details of the conditional logistic regression for the most frequent patterns when applying a one‐year time‐lag.
**Figure S4.** Most frequent patterns of abiraterone and enzalutamide exposure.
**Figure S5.** Patterns of earliest and longest exposure to abiraterone and enzalutamide.
**Figure S6.** Estimated odds ratios with 95% confidence intervals according to the weighted cumulative exposure method.

**Data S1.** Supporting Information

**Data S2.** Supporting Information

## Data Availability

For legal reasons, access to the data (SNDS) is not public but reserved for individual authorizations, validated by the national health insurance fund (CNAM). However, the analysis codes (or scripts) may be shared with anyone who submits a reasoned request.
