# Supplementary material for: Second primary cancers and hormonal therapies for prostate cancer: A nested case–control study
Source: Fundam Clin Pharmacol. 2025 Mar 17;39(2):e70004. doi: 10.1111/fcp.70004 (PMC11913542; doi:10.1111/fcp.70004)
Supplement: Supplementary file 1 — Table S1. Details of codes. Table S2. Details of the second primary cancer codes (n = 8038) by decreasing frequency. Table S3. Conditional odds ratios for second primary cancer stratified by drug cumulative exposure. Figure S1. Case–control eligibility. A – for cases. B – for controls. Figure S2. Drug exposure group among eligible cases (in the population not switching ARPI). Figure S3. Details of the conditional logistic regression for the most frequent patterns when applying a one‐year time‐lag. Figure S4. Most frequent patterns of abiraterone and enzalutamide exposure. Figure S5. Patterns of earliest and longest exposure to abiraterone and enzalutamide. Figure S6. Estimated odds ratios with 95% confidence intervals according to the weighted cumulative exposure method. [file FCP-39-0-s002.docx]

**Appendices**

**Table S1. Details of codes**

**Table S2. Details of the second primary cancer codes (n = 8038) by decreasing frequency**

**Table S3. Conditional odds ratios for second primary cancer stratified by drug cumulative exposure.**

**Figure S1. Case-control eligibility**

**A – for cases**

**B – for controls**

**Figure S2. Drug exposure group among eligible cases (in the population not switching ARPI)**

**Figure S3. Details of the conditional logistic regression for the most frequent patterns when applying a one-year time-lag.**

**Figure S4. Most frequent patterns of abiraterone and enzalutamide exposure**

**Figure S5. Patterns of earliest and longest exposure to abiraterone and enzalutamide**

**Figure S6. Estimated odds ratios with 95% confidence intervals according to the weighted cumulative exposure method**

**Appendix Table S1.**

| **Variable label** | **Definition** | **Length of history** | **ICD-10 codes for hospitalisation or Long-term/chronic diseases (LTD)** | **Medical act code** | **ATC code** | **Biology codage** |
| --- | --- | --- | --- | --- | --- | --- |
| History of other active cancer | Active cancer other than Prostate cancer identified before T0 | From 2009 up to cohort entry (T0) | C00 –C96, excluding C61 (prostate cancer) and C44 (Other malignant skin neoplasms) ;  D00-D09 excluding D075, D400, D291 |  | L01Axx, L01BA03-04, L01BB, L01BCxx, L01Cxx, L01Dxx, L01EAx, L01EBx, L01ECx, L01EDxx, L01EE-01 to 03, L01EFxx, L01EGxx, L01EHxx, L01EJxx, L01EKxx, L01ELxx, L01EMxx, L01ENxx, L01EX-01 to 08 L01EX-10 to 23, L01FA03, L01FBxx L01FCxx, L01FDxx, L01FExx, L01FFxx, L01FGxx, L01Fxx, L01XAxx, L01XAxx, L01XBxx, L01XD04, L01XFx, L01XGxx, L01XHx, L01XKxx, L01XX-01 to 03 and L01XX08, L01XX11, L01XX-23 -24 -27 -29 -40 -41 -44 -52 -53 -59 -62 -66 -69 -70 -71 -73, L01XYxx, L02AB01, L02BAxx, L02BGxx, L03AC01, L03AX11, L04AA34, L04AX04, L04AX06, L01XX35, L04AX02, L01EJxx |  |
| Second primary cancer |  | from T0 to end of December 2021 | C00-C96, not considering C61 (prostate cancer) nor C44 (other malignant skin neoplasms), and  excluding some cases with C77-C80 or D00-D48 codes  (see appendix Figure S1) |  |  |  |
| PSA dosage |  |  |  |  |  | 7318, 7319, 7320 |
| ADT treatment | GnRH agonist or antagonist,  1st generation of antiandrogen, orchiectomy |  |  | JHFA010 | L02AExx, L02BX02, L02BBxx, G03HAxx |  |
| ABI treatment | Abiraterone exposure |  |  |  | L02BX03 |  |
| ENZ treatment | Enzalutamide exposure |  |  |  | L02BB04 |  |
| APA treatment | Apalutamide exposure |  |  |  | L02BB05 |  |
| Darolutamide treatment | Darolutamide exposure |  |  |  | L02BB06 |  |
| Prostatectomy | History of prostatectomy | With retrospect up to 2009 |  | JGFC001, JGFA006, JGFA011, JDFA019, JDFA020, JDFA021, JDFA022, JDFA023, JDFA024, JDFA025 |  |  |
| Radiotherapy Curietherapy | History of radiotherapy sessions  or event/complications due to radiotherapy sessions | With retrospect up to 2009 | Y63.2, Z08.1, Z09.1, Z51.0,  Z54.1, Z92.3, N30.4, K52.0,  L58, L59, J700, T66, K627, G628, G958, G938, M962, M965, |  |  |  |
| Myocardial infarction |  | Within 3 years before the cohort entry | I21.x |  |  |  |
| Ischemic heart disease |  | Within 3 years before the cohort entry | I20.x, I21.x, I22.x, I23.x, I24.x, I25.x |  |  |  |
| Congestive heart failure |  | Within 3 years before the cohort entry | I110, I130, I132, I50.x |  |  |  |
| Peripheral vascular disease |  | Within 3 years before the cohort entry | I70.x, I71.x, I731.x, I738.x, I739.x, I771.x, I790.x, I792.x, K551.x, K558.x, K559.x Z958.x, Z959.x | EEAF.x, ECPF.x, ECFA.x, EBFA.x,, EENF.x, EEPF.x, EEFA.x, EECA.x, ENNF.x, ENFA.x, DGAF.x, EDAF.x, DGFP.x, EDPF.x, DGFA.x, EDFA.x, DGCA.x, EDCA.x, EDPF.x, EDJF.x, EDKA.x, EDEA.x, EDLF.x, EDNF.x, EDPF.x, EDJF.x, EDMA.x, EANF002, ECNF002, ECJF001, ECCA007, ECCA009, ECCA003, ECCA002, ECMA001, ECKA002, EBNF001, EDEA001, EDLF007, EEJF001, EDCA005, ENAF001, ENAF002, DGKA004, EDNF003, EDKA002 |  |  |
| Cerebrovascular disease |  | Within 3 years before cohort entry | G45.x, G46.x, H34.0, I60.x - I69.x |  |  |  |
| Dementia |  | Within 3 years before cohort entry | F00.x–F03.x, F05.1, G30.x, G31.1 |  | N06D%  *(at least 3 reimbursements)* |  |
| Chronic pulmonary disease |  | Within 3 years before cohort entry | I27.8, I27.9, J40.x–J47.x, J60.x–J67.x, J68.4, J70.1, J70.3 |  | R03%  except R03AA, R03AB, R03CA, R03CB  *(at least 3 reimbursements)* |  |
| Rheumatologic disease Connective tissue disease |  | Within 3 years before cohort entry | M05.x, M06.x, M315, M32.x-M34.x, M351, M353, M360 |  |  |  |
| Peptic ulcer disease Ulcer disease |  | Within 3 years before cohort entry | K25.x-K28.x |  |  |  |
| Mild liver disease |  | Within 3 years before cohort entry | B18.x, K70.0–K70.3, K70.9, K71.3–K71.5, K71.7, K73.x, K74.x, K76.0, K76.2–K76.4, K76.8, K76.9, Z94.4 |  |  |  |
| Moderate or severe liver disease |  | Within 3 years before cohort entry | I85.0, I85.9, I86.4, I98.2, K70.4, K71.1, K72.1, K72.9, K76.5-K76.7 |  |  |  |
| Diabetes Without complications |  | Within 3 years before cohort entry | E100, E101, E106, E108, E109, E110, E111,E116, E118, E119, E120, E121, E126, E128,E129, E130, E131,E136, E138, E139, E140, E141, E146, E148, E149 |  | A10%  *(at least 3 reimbursements)* |  |
| Diabetes with end-organ damage |  | Within 3 years before cohort entry | E102-E105, E107, E112-E115, E117, E122-E125, E127, E132-E135, E137, E142-E145, E147 | BGNA001, BGNP001, BGNP004, BGNP006, BGNP007, BGNP008 |  |  |
| Hemiplegia |  | Within 3 years before cohort entry | G04.1, G11.4, G80.1, G80.2, G81.x, G82.x, G83.0–G83.4, G83.9 |  |  |  |
| Moderate or severe renal disease |  | Within 3 years before cohort entry | I12.0, I13.1, N03.2–N03.7, N05.2–N05.7, N18.x, N19.x, N25.0, Z49.0–Z49.2, Z94.0, Z99.2 | JVJF004, JVJF008, JVJB001, JVJF003 |  |  |
| AIDS/HIV |  | Within 3 years before cohort entry | B20.x–B22.x, B24.x, Z21.x |  |  |  |

**Table S2.**

Cancers in potentially previously irradiated areas

N = 1917 (24%)

| **Cancer type by system organ class** | **Frequency** | **Percent (%)** |
| --- | --- | --- |
| Digestive organs   - Non pelvic - Pelvic   *Malignant neoplasm of the rectum*  *Malignant neoplasm of the rectosigmoid junction*  *Malignant neoplasm of the anus and anal canal* | 2799  2254  545  *387*  *113*  *45* | 34.8  28.0  6.8  *4.8**  *1.4**  *0.6** |
| Urinary tract   - Non bladder genitourinary tract - Bladder genitourinary tract   *Malignant neoplasm of bladder*  *Malignant neoplasm of ureter* | 1739  359  1380  *1337*  *43* | 21.9  4.5  17.4  *16.9**  *0.5** |
| Bronchus or lung | 1023 | 12.7 |
| Lymphoid tissue | 518 | 6.4 |
| Melanoma | 422 | 5.3 |
| Hematopoietic tissue | 407 | 5.1 |
| Lip, oral cavity and pharynx | 320 | 4.0 |
| Respiratory and intrathoracic organs (non-lung) | 209 | 2.6 |
| Central nervous system including eye | 161 | 2.0 |
| Mesothelial and soft tissue | 97 | 1.2 |
| Male genital organs** | 85 | 1.1 |
| Bone and articular cartilage | 88 | 1.1 |
| Endocrine glands | 69 | 0.9 |
| Breast | 53 | 0.7 |
| Malignant tumor of other or undefined sites | 19 | 0.2 |
| Total | 7928 | 100 |

* The proportion of cancer subtypes was obtained by dividing the number of individuals of a given cancer by the total number of eligible cases (n = 8038).

**Including of penis and of testis.

**Table S3. ﻿**

| Cumulative drug exposure* | Number of cases/controls | Odds ratios (95% Confidence interval) |
| --- | --- | --- |
| ARPI non-user | 7728/77219 | 1.0 |
| < 1 year |  |  |
| ABI | 83/538 | 1.5 (1.2-2.0) |
| ENZ | 57/400 | 1.4 (1.1-1.9) |
| 1 – 2 years |  |  |
| ABI | 30/149 | 2.1 (1.4-3.0) |
| ENZ | 17/140 | 1.2 (0.7-2.0) |
| 2 – 3 years |  |  |
| ABI | 8/34 | 2.3 (1.1-5.1) |
| ENZ | 3/43 | 0.7 (0.2-2.3) |
| > 3 years |  |  |
| ABI | 2/12 | 1.7 (0.4-7.4) |
| ENZ | 0/19 | Not estimated |

ABI: abiraterone; ENZ: enzalutamide

* A one-year time-lag was applied, not taking into account the year of exposure before the case date and matched-controls.

**Figure S1A.**

**
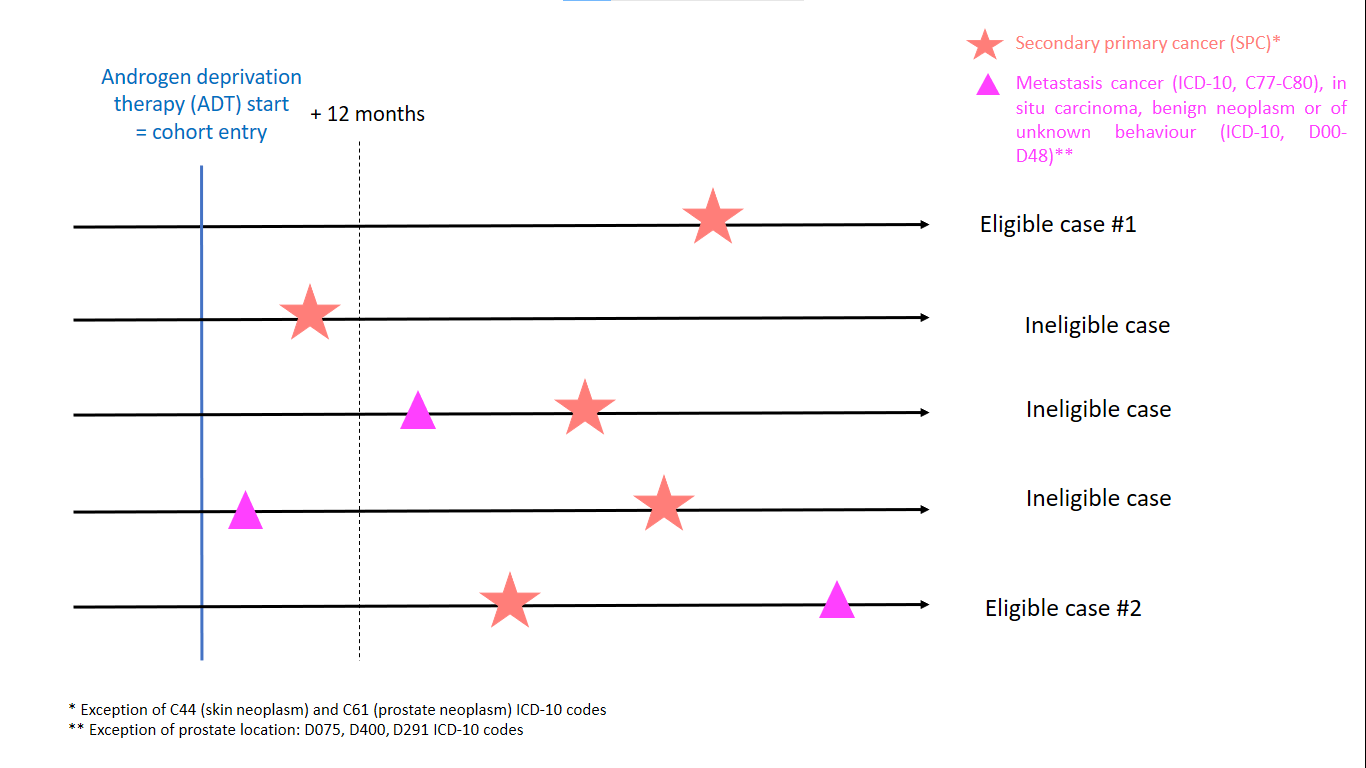
**

**Figure S1B.**

**
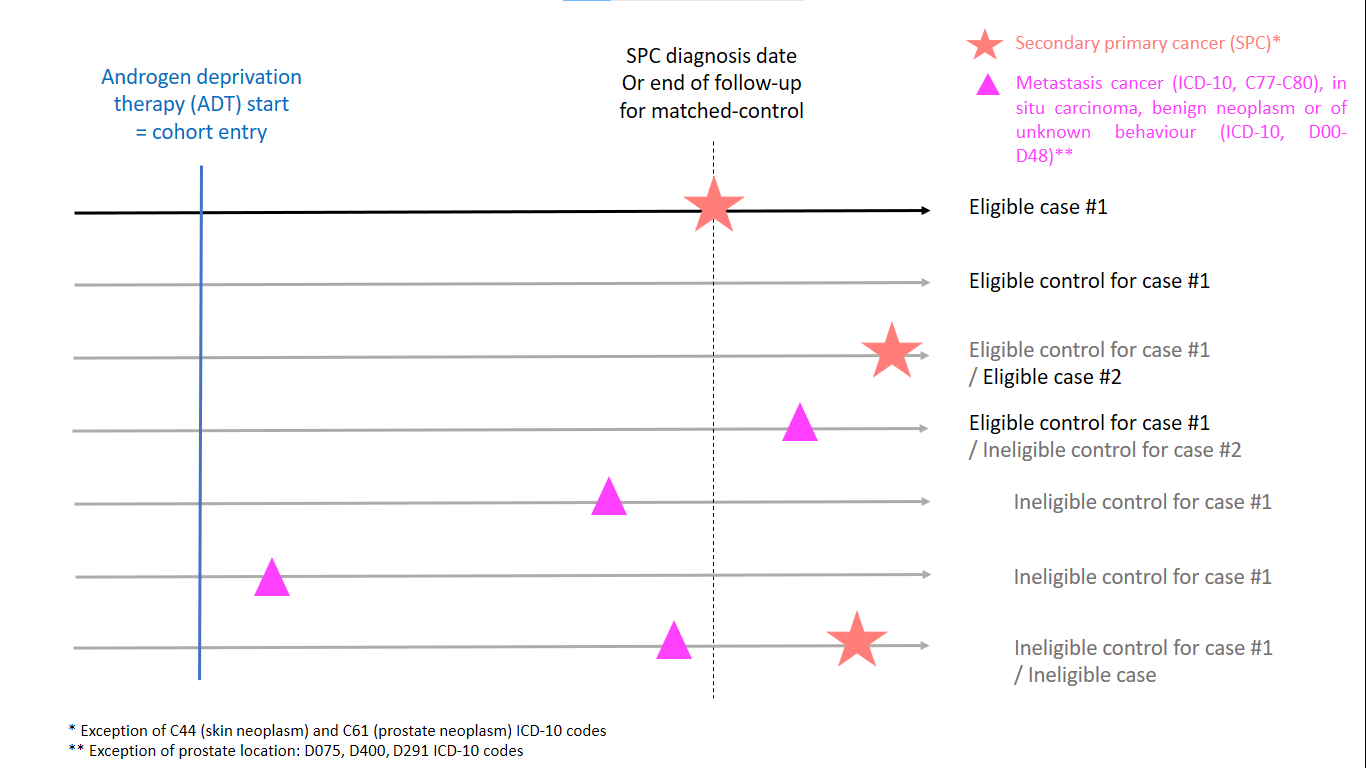
**

**Figure S2.**

**
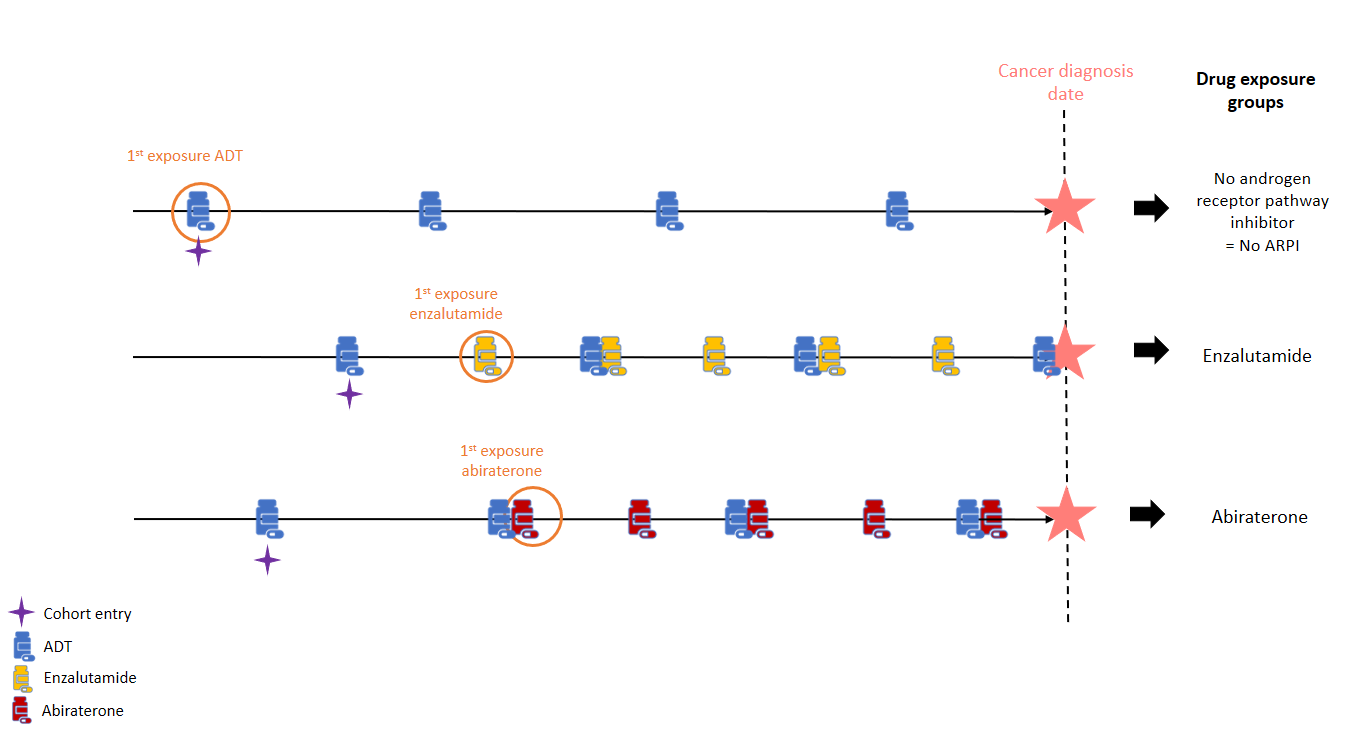
**

**Figure S3.**

Enzalutamide exposure

3 years or more

Year before the SPC diagnosis date

| ***Pattern*** | ***Nb of patients*** | ***Nb of cases*** | ***Nb of controls*** | ***OR^£^*** | ***IC 95%*** |
| --- | --- | --- | --- | --- | --- |
| Line 1 | 228 | 33 | 195 | 1.68 | 1.16 - 2.44 |
| Line 2 | 114 | 13 | 101 | 1.29 | 0.72 - 2.30 |
| Line 3 | 63 | 4 | 59 | 0.67 | 0.24 - 1.84 |
| Line 4 | 35 | 5 | 30 | 1.65 | 0.64 - 4.26 |
| Line 5  (3 years or more) | 50 | 3 | 47 | 0.64 | 0.20 - 2.07 |


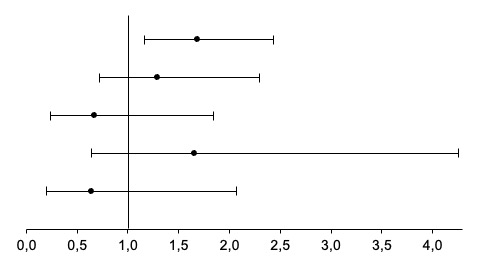

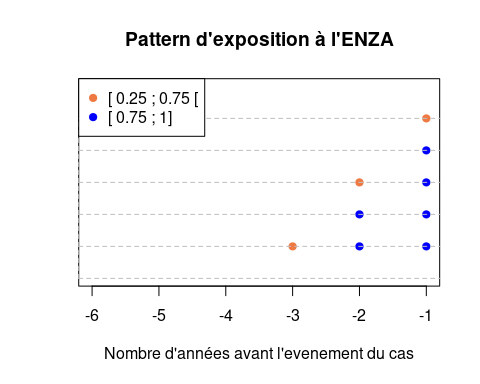


Partial annual exposure

Full annual exposure

3 years or more

Year before the SPC diagnosis date*

Odds ratio with 95% confidence interval

Abiraterone exposure

3 years or more

Year before the SPC diagnosis date

| ***Pattern*** | ***Nb of patients*** | ***Nb of cases*** | ***Nb of controls*** | ***OR^£^*** | ***IC 95%*** |
| --- | --- | --- | --- | --- | --- |
| Line 1 | 263 | 30 | 233 | 1.29 | 0.88 - 1.89 |
| Line 2 | 173 | 26 | 147 | 1.78 | 1.17 - 2.71 |
| Line 3 | 76 | 14 | 62 | 2.26 | 1.26 - 4.03 |
| Line 4 | 58 | 8 | 50 | 1.62 | 0.77 - 3.42 |
| Line 5  (3 years or more) | 52 | 7 | 45 | 1.55 | 0.70 - 3.44 |


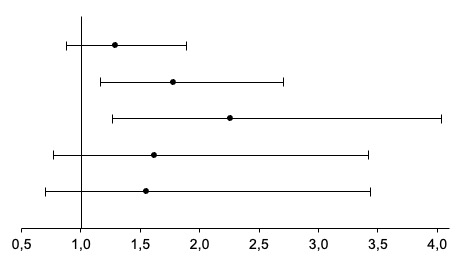

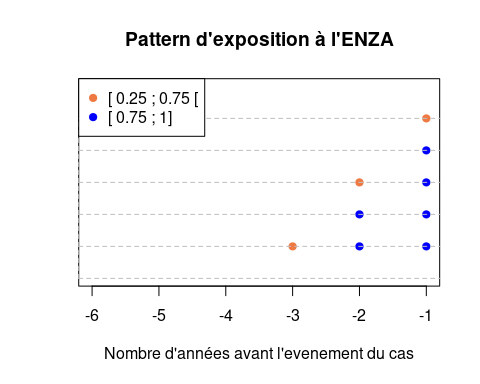


Partial annual exposure

Full annual exposure

3 years or more

Year before the SPC diagnosis date*

Odds ratio with 95% confidence interval

*A one-year time-lag was applied, not considering the year of drug exposure before the SPC diagnosis date.

*^£^The* reference was the ARPI non-user group.

**Figure S4**

**S4.A. Most frequent exposure to enzalutamide S4.B. Most frequent exposure to abiraterone**

Nb of patients

532

210

177

146

63

51

46

35

23


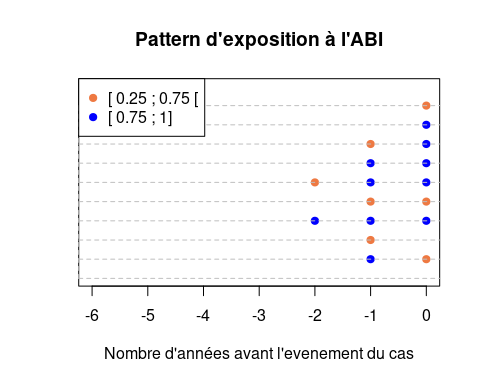

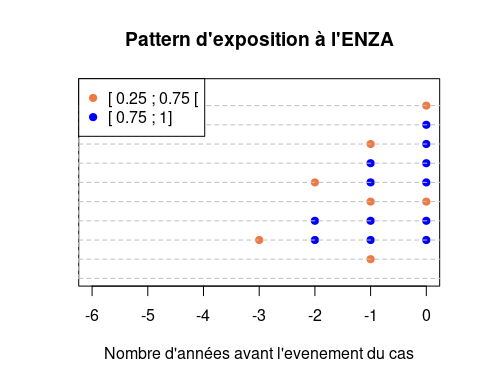


Partial annual exposure

Full annual exposure

Partial annual exposure

Full annual exposure

Year before the SPC diagnosis date

(0 is the year of the cancer diagnosis)

Nb of patients

478

178

163

95

49

44

30

25

21

Year before the SPC diagnosis date

(0 is the year of the cancer diagnosis)

**Figure S5.**

**S5.A. Earliest and longest exposure to enzalutamide S4.B. Earliest and longest exposure to abiraterone**

Nb of patients

4

3

1

1

1

1

1

1

1

1

Nb of patients

7

5

3

1

1

1

1

1

1

1


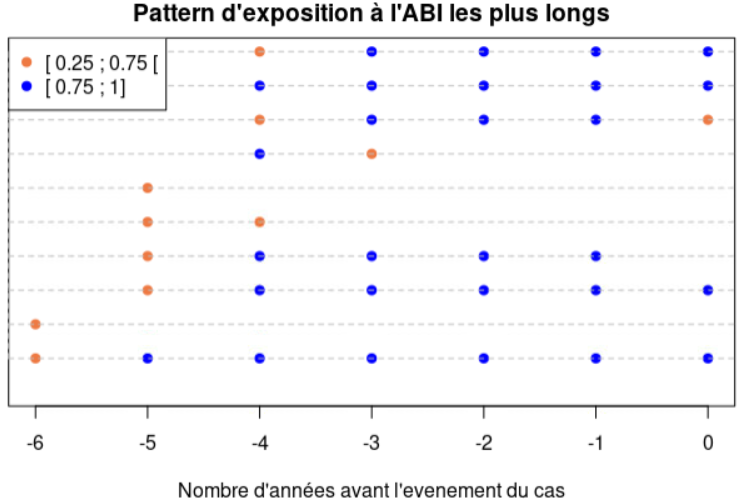

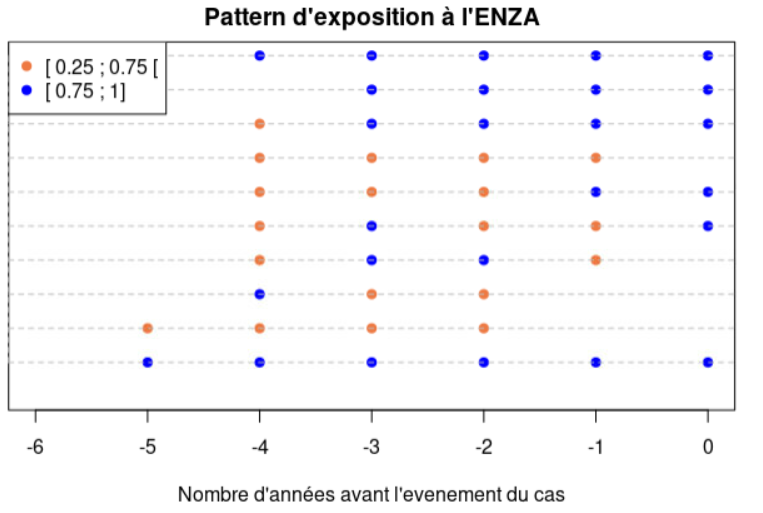


Partial annual exposure

Full annual exposure

Partial annual exposure

Full annual exposure

Year exposure before the SPC diagnosis date

(0 is the year of the cancer diagnosis)

Year exposure before the SPC diagnosis date

(0 is the year of the cancer diagnosis)

**Figure S6.**

The WCE model took into account only 6 years (the current year of the SPC date + 5 years before) for enzalutamide exposure (left graph) and 5 years (the current year of the SPC date + 4 years before) for abiraterone exposure (right graph).

**WCE for enzalutamide exposure WCE for abiraterone exposure**


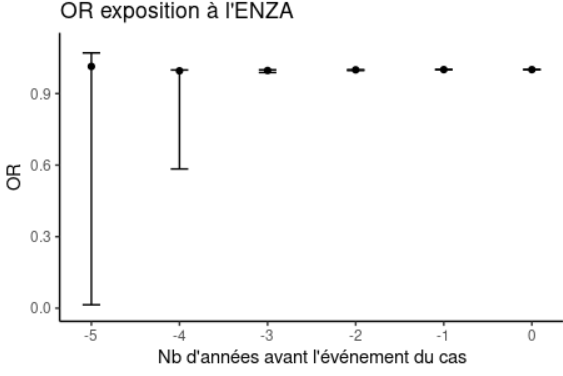

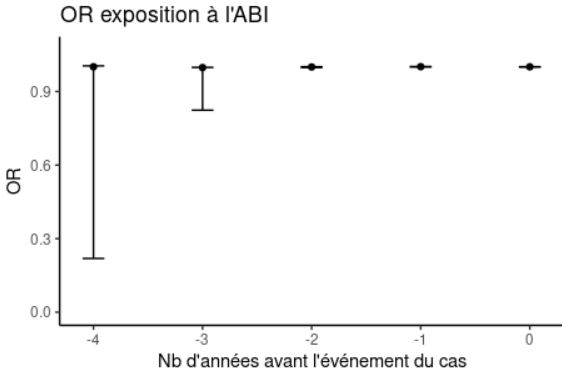


OR: odds ratio
