# Supplementary material for: Second primary cancers and hormonal therapies for prostate cancer: A nested case–control study
Source: Fundam Clin Pharmacol. 2025 Mar 17;39(2):e70004. doi: 10.1111/fcp.70004 (PMC11913542; doi:10.1111/fcp.70004)
Supplement: Supplementary file 3 — Data S2. Supporting Information [file FCP-39-0-s003.docx]

| **Title** | Second cancers and hormonal therapy in prostate cancer: a French population-based study |
| --- | --- |
| **Acronym** | SCHNAPs (Second Cancers and HormoNAl therapy in Prostate cancer) |
| **Date of last version of protocol** | 09 August 2021 |
| **Active substance** | LHRH agonists  LHRH antagonist  Enzalutamide  Abiraterone Acetate |
| **Research question and objectives** | Second generation of hormonal therapies for advanced prostate cancer may be associated with increased risk of second primary cancer.  The primary objective is to investigate the impact of enzalutamide or abiraterone exposure on subsequent risk of developing secondary primary cancer compared to androgen deprivation therapy alone among prostate cancer patients. |
| **Country of study** | France |
| **Author &**  **Principal investigator** | Lucie-Marie SCAILTEUX  EA-7449 REPERES, University of Rennes 1  Lucie-marie.scailteux@univ-rennes1.fr |

# 2. List of abbreviations

ADT Androgen deprivation therapy

ATC Anatomical Therapeutic Chemical classification system

BMD Bone mineral density

CRPC Castration resistant prostate cancer

ICD International Classification of Diseases

IMRT Intensity-modulated radiation therapy

OR Odds ratio

PSA Prostate-specific antigen

RP Radical prostatectomy

RT Radiation therapy

**3. Milestones**

| Milestone | Planned date |
| --- | --- |
| DRUG-SAFE^®^ and EPI-PHARE validation | September 31^st^, 2021 |
| Start of data collection | December 1^st^, 2021 |
| End of data collection | January 15^th^, 2022 |
| Final report of study results | April 30^th^, 2022 |

# 4. Rationale and background

The global burden of prostate cancer is substantial, ranking among the top five cancers for both incidence and mortality. Prostate cancer is the most commonly diagnosed cancer in men.

Whereas some men have an aggressive form of prostate cancer, most others have a slow-growing or indolent form of disease. Although most men are diagnosed with localized prostate cancer.

Between 25 and 50 % of all patients undergoing radical prostatectomy (RP) or radiation therapy (RT) develop a rising prostate-specific antigen (PSA) level (PSA recurrence)^1^. Biochemical recurrence after RP or RT precedes clinical metastases by 7–8 year on average. The standard of care for patients with locally advanced prostate cancer (T3–4 N0, M0) is intensity-modulated radiation therapy (IMRT) combined with long-term androgen deprivation therapy (ADT) for at least 2–3 years. An increased risk of second primary cancers have been observed in the irradiated areas^2^.

Still around 20% of men are diagnosed with advanced disease and require hormonal therapies (Figure-1 in Annex). Hormonal therapies are used to delay the progression to metastatic prostate cancer or extend overall survival of patients with metastatic prostate cancer.

Enzalutamide (Xtandi®) and Abiraterone (Zytiga®) are used since early 2010s in France in the treatment of **metastatic castration resistant prostate cancer (mCRPC) before / after docetaxel** (based on the results of the AFFIRM and PREVAIL trials for enzalutamide, and COU-AA-301 and COU-AA-302 for abiraterone), with a first market authorization in 2011 for abiraterone and 2013 for enzalutamide. These drugs were used before the market authorization (“ATU” status) in 2010-2011 for abiraterone and in 2013 for enzalutamide, in addition to ADT. In France between 2014-2017, about 10 300 mCRPC patients were new users of abiraterone and enzalutamide; median overall survival varied between 32 to 34 months.^3^

New indication of enzalutamide in **non-metastatic (M0) castration resistant prostate cancer (nmCRPC)** has been approved in 2018 in Europe (based on the PROSPER trial). The market use is authorized in France since March 2020. In this indication, apalutamide and darolutamide could also be used (based on the SPARTAN and ARAMIS trials, respectively) in addition to ADT, respectively launched in France in March 2020 and March 2021. Enzalutamide and apalutamide are very similar in structure and functions. Both are selective Androgen Receptor (AR) inhibitor that binds directly to the ligand-binding domain of the AR, preventing AR nuclear translocation, inhibiting DNA binding, impeding AR-mediated transcription, and lacking androgen receptor agonist activity.

New indication of abiraterone **in metastatic castration-sensitive prostate cancer (mCSPC)** has been approved in late 2017 in Europe (based on the results of the LATITUTE and STAMPEDE trial). French commercialization was authorized in February 2019. Enzalutamide and apalutamide has been shown to improve clinical outcomes in mCSPC, in TITAN (apalutamide) and ENZAMET and ARCHES (enzalutamide) trials. European market authorizations were obtained in December 2019 (apalutamide) and May 2021 (enzalutamide), and they are not yet commercialized in France in this indication.

Regardless the prostate cancer stage and the treatment indication, the use of androgen deprivation therapy (ADT; GnRH agonist or antagonist) is recommended in association with abiraterone and enzalutamide (and apalutamide). It should be noted that before the LATITUDE trial^4^, ADT alone (including GnRH agonist and antagonist +/- antiandrogen) was the standard of care for locally advanced to hormone-sensitive metastatic diseases^5^ (figure 1). Among ADT, orchiectomy can be marginally used^6^ to induce a permanent testosterone deprivation. According to the STAMPEDE trial, median survival of treated patients with newly diagnosed metastases was approximately 42 months with ADT monotherapy^7^.


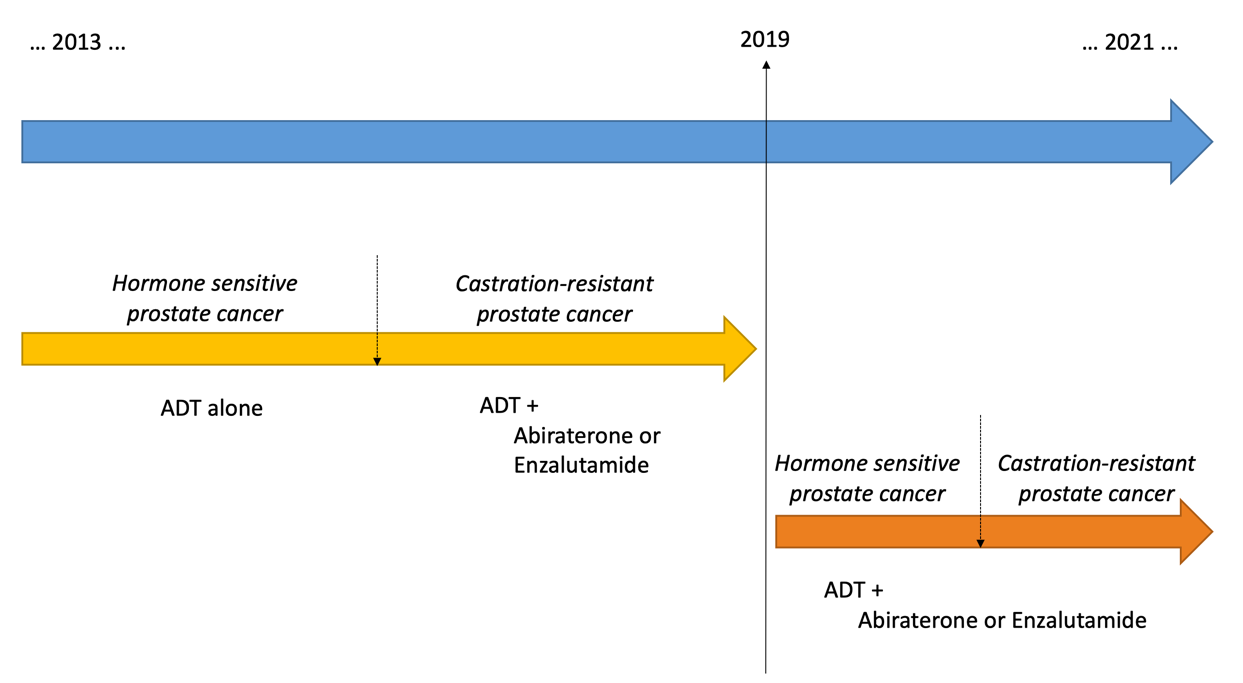


**Figure 1. Change in French prostate cancer guidelines.**

Currently, the prostate cancer treatment timeline is explained in Figure 2 according to Vellky et Rycke^8^.


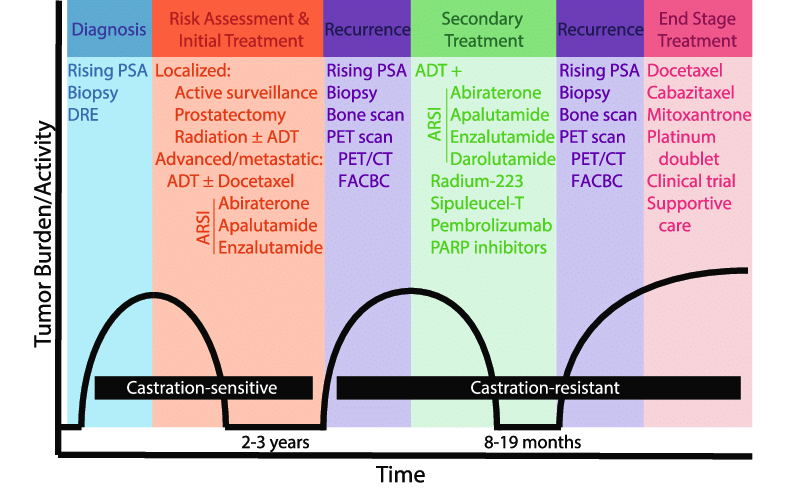


# Figure 2. Prostate Cancer Progression to CRPC Timeline and Treatments. Schematic for diagnosis and treatment of CaP through progression to CRPC. DRE = digital rectal exam, PSA = prostate-specific-antigen, ADT = androgen deprivation therapy (LHRH agonist/antagonists), ARSI = androgen receptor signaling inhibitor, abiraterone = abiraterone acetate, PET = positron emission tomography, CT = computed tomography, FACBC = anti-1-amino-3-18F-fluorocyclobutane-1-carboxylic acid (also known as fluciclovine F18), PARP = Poly (ADP-ribose) polymerase

At the time of mCRPC, switch could be observed^9,10^. In a previous French cohort study of 10 300 patients mCRPC patients (the ‘SPEAR’ cohort^3^), between 2014 and 2018, about 10 and 20 % of new users of abiraterone and enzalutamide, respectively, switched to enzalutamide / abiraterone or chemotherapy in the first 12 months of treatment (data not shown).

## 4.1. Safety hazard(s), safety profile or risk management measures

The safety profile of enzalutamide concerned neurological, cardiac and general disorders.

The risk management plan (last updated May 12^th^, 2021) of Xtandi® (enzalutamide) mentioned among important identified risks seizure, fall, non-pathological fracture and ischemic heart diseases^11^. No important potential risk or missing information are mentioned.

Due to its pharmacological mechanism, the safety profile of abiraterone included cardiac disorders, especially cardiac failure as well as hypertension, hypokalaemia and fluid retention, which motivate a monitoring of the cardiac function. Hepatotoxicity also belong to the most expected ADR^12^.

The risk management plan (from the last assessment report variation in 2017) of Zityga® (abiraterone) mentioned hepatotoxicity, cardiac disorders, osteoporosis (and related fractures), rhabdomyolysis/myopathy, and allergic alveolitis^13^. Anemia, cataract, and drug-drug interaction (CYP 2D6) belong to the potential important risks.

With the increased exposure to those abiraterone and enzalutamide, especially at less advanced stages, patients may be at an increased risk for developing second primary malignancies (see section 4.2).

## 4.2. Critical review of available data

### **4.2.1. Animal and human experiments**

For **Enzalutamide** carcinogenicity data with daily dosing of rats for two years produced an increased incidence of neoplastic findings. Benign Leydig cell tumours in the testes, urothelium papilloma and carcinoma of urinary bladder in males were the most prominent neoplastic findings; thymoma, adenoma in the pars distalis of the pituitary were also observed^14^. The human relevance of thymoma, and pituitary adenoma as well as urothelium papilloma and carcinoma of urinary bladder cannot be ruled out. Urothelial papilloma/carcinoma in the urinary bladder could be induced by continuous local irritation of the epithelium by crystals or calculi that consist of excreted carboxylic acid metabolite^14^. Calculi and crystals were observed in rat urinary bladders.

**Abiraterone** acetate was not carcinogenic in a 6-month study in the transgenic (Tg.rasH2) mouse. In a 24-month carcinogenicity study in the rat, abiraterone acetate increased the incidence of interstitial cell neoplasms in the testes^15^. This finding is considered related to the pharmacological action of abiraterone and rat specific.

### **4.2.2. Clinical studies**

The **abiraterone** clinical trials, COU-AA-301, COU-AA-302 and LATITUDE, mentioned the occurrence of neoplasm (benign, malignant or unspecified) in up to 12 cases in the abiraterone arms, without details on the localisation^16,17^.

Among phase III **enzalutamide** trials, second primary malignancies (excl. non melanoma skin cancer) were observed in 3% of the enzalutamide arms^14^. The PROSPER trial reported 48 (5%) second primary cancer in the enzalutamide group (N = 930) (including 7 cases [brain neoplasm, acute myeloid leukemia, small-cell lung cancer…] leading to death) versus 7 (2%) in the placebo group^18^. According to the 2021 EMA assessment report, the potential risk of enzalutamide to develop second primary malignancies, especially urinary bladder cancer, cannot be ruled out^14^ (see animal experiments section above).

In phase 3 clinical studies, the most frequently reported events in **enzalutamide** treated patients, and greater than placebo, were bladder cancer (0.3%), adenocarcinoma of the colon (0.2%), transitional cell carcinoma (0.2%) and bladder transitional cell carcinoma (0.1%)^19^.

### **4.2.3. Epidemiologic studies**

A population-based study conducted in Taiwan between 2000 and 2010 showed a crude SPC incidence of 13.5 per 1000 person-years in a **prostate cancer cohort**^20^. No information was provided regarding the distribution of cancers by stage of prostate cancer or treatments used. Through a 1999-2011 Swedish population-based study, crude incidence rate of SPC was 82 per 1000 patients-years (95% CI, 79-85) for **metastatic prostate cancer** and 116 per 1000 patients-years (95% CI, 95-141) for **mCRPC** (both cohorts included patients with a history of malignant neoplasms and secondary malignancies)^21^.

So far, no study assessed the risk of secondary primary cancer in prostate cancer patients exposed to abiraterone or enzalutamide. Few epidemiological data are available on the risk of second cancer among CRPC patients.

Conducting a retrospective cohort study on 2,234 men aged ≥ 65 years with **CRPC** (84.5% had bone metastases) identified in the Surveillance, Epidemiology, and End Results (SEER)-Medicare database from January 1, 2000, to December 31, 2013, authors observed second primary cancer in 172 patients (incidence rate 59 per 1000 person-years)^22^. The most common cancers concerned lung/bronchus (n = 29, 16.9%), urinary bladder (n = 22, 12.8%), and colon/rectum (n = 21, 12.2%). The incidence rate was approximately threefold higher (standardized incidence ratio = 3.1, 95% CI, 2.8-3.6) than the population-based cancer incidence among men without prostate cancer. **Docetaxel** was the most frequently identified (76%) second-line systemic CRPC therapy, followed by **abiraterone** (9.6%) and **sipuleucel-T** (8.5%).

Conducting a retrospective cohort study based on the German Pharmacoepidemiological Research Database (GePaRD), the Prostate Cancer Database Sweden (PCBaSe), and the SEER database, among 2360, 2849, and 2234 men with **mCRPC** and new users of second line systemic therapies (**abiraterone, enzalutamide, docetaxel, cabazitaxel**…), respectively, incidence of second primary malignancies were 79 (95% CI: 70.4–88.4), 102 (95% CI: 90.3–114.5) and 59 (95% CI: 50–68) per 1000 person-years in German, Swedish and US cohorts, respectively^23^. The most observed site-specific concerned urinary bladder, lung and colon-rectum. No information was provided regarding the repartition of cancer among second line systemic therapies.

## 4.3. Expected contribution of the current study

# Available data have suggested a risk of second primary cancer in prostate cancer patients, and a potential risk of carcinogenicity from hormone therapies cannot be excluded. As these drugs are now indicated at an earlier stage of the disease, it is important to assess the risk in a dedicated study. If it were to be considered a new ADR, it could be a concurrent event with prostate cancer death and change recommendations for prostate cancer treatment.

# 5. Research question and objectives

## 5.1. Research question

Are second-generation androgen receptor antagonists, particularly abiraterone and enzalutamide, associated with an increased risk of second primary cancer (SPC) in prostate cancer survivors?

## 5.2. Primary objective

### To compare the risk of SPC among prostate cancer survivors newly exposed to either enzalutamide, abiraterone, or androgen deprivation therapy alone.

**6. Research methods**

## 6.1. Study design

### **6.1.1. Overall research design**

We designed a population-based nested case-control study using the French National Health Data System. (‘SNDS’) on the 2009-2021 period. The cohort will encompass all new users of ADT used for prostate cancer in the period 2013-2019 and followed up to December 2021. History of patients will be identified from January 2009. We will secondly identify the subsequent use of abiraterone and enzalutamide in association with ADT. Case will be patients with a diagnosis a SPC and controls will be matched patients without SPC identified. Given the time required to load the SNDS database and to validate the data, at the time of the analyses, we plan an initial analysis using both consolidated and unconsolidated data (especially for the year 2021). Then, the study will be replicated when the data are all consolidated.

### **6.1.2. Rationale for this choice**

When studying exposures that vary with time (add-on of new hormonal therapy [abiraterone, enzalutamide] on top of ADT when prostate cancer becomes CRPC, and subsequent switch between hormonal therapies and chemotherapy [docetaxel, cabazitaxel]), an additional level of complexity is introduced by the need to account for time-dependent exposure in both the design and analysis. The nested case-control approach is a useful alternative for cohort analysis when studying time-dependent exposures^24^. Its superior computational efficiency may be particularly useful when studying rare outcomes in databases^25^. A potential advantage with respect to design is the option to match controls to cases on the basis of possible confounding covariates for which estimation of effect is not of interest. The nested case-control approach allows to address the possibility that the effect of the exposure varies over time by analysing latency-weighted exposures^26^.

## 6.2. Data sources

The study is based on the French National Health Data System (‘SNDS’, *Système National Des Données De Santé*). ‘SNDS’ links the healthcare reimbursement database to the French hospital discharge database. The database collects since early 2010’s all claims covered by one of the several French insurance plans (99% of the French population). The database contains all reimbursements of drugs (with date of dispensing, quantity of tablets, dose), and vital status. It also contains the long-term chronic disease status, allowing for full medical reimbursement of health expenditure related to chronic costly disease (with starting date and medical diagnosis, ICD-10). The hospital discharge database provides diagnoses through ICD-10 codes with entry date^27,28^.

### **6.3. Cohort selection criteria**

**6.3.1. Inclusion and exclusion criteria**

As a reminder, before 2019 and the market authorization of abiraterone in hormone-sensitive prostate cancer, only ADT were used (see section 4). Over our 2013-2021 study period, several schemes of hormonal treatment could be observed (see figure 1 above):

- as a proxy of hormone sensitive prostate cancer (metastatic or not):

- patients with ADT alone (before 2019)

- patients with ADT with a concomitantly introduced abiraterone (after 2019)

- as a proxy of mCRPC: patients with ADT and a subsequent addition of abiraterone or enzalutamide (since 2014-2015)

The cohort will encompass:

- new users of ADT (Luteinising hormone-releasing hormone agonists (Leuprorelin, Triptorelin, Goserelin…; ATC L02AE%), or antagonists (Degarelix; ATC L02BX02) for prostate cancer (ICD-10 code for chronic disease “*ALD, affection longue durée”* C61 – prostate cancer) with a first reimbursement (code list in Annex 1) between January, 2013 and December, 2021,

- having or not a concomitant hormonal therapy (enzalutamide, ATC L02BB04 or abiraterone, ATC L02BX03)

- with no reimbursement of ADT observed in 2011-2012, including also all anti-androgens (anti-androgen of 1^st^ generation: ATC codes L02BB, G03HA) and orchiectomy: medical act JHFA010).

Patients with another primary cancer than prostate cancer identified between January 2009 and date of the ADT initiation (except for non-melanoma skin cancer) will be excluded. This will be based on long-term chronic disease status, along with diagnosis ICD-10 code (C00-C76, C81-C96, excluding C61) and the starting date, outpatient anticancer drugs (of course different from those used in prostate cancer), chemotherapy or radiotherapy sessions with a related diagnosis of cancer different from prostate cancer or bone metastasis, and primary or related or associated hospital discharge diagnosis of cancer different from prostate cancer or bone metastasis.

**6.4 Nested case-control study**

### **6.4.1. Case and control definition**

Case will be patients with a SPC identified between January 2013 and December 2021. Incident SPC will be identified through ICD-codes C00-C76, C81-C96, excluding C61. Accuracy of the algorithms to estimate cancer incidence have been already assessed^29^.

Controls will be patients without SPC cancer at the SPC date of the case. Up to 10 controls will be randomly selected from the risk-set of each case (i.e. subjects present in the cohort at the time the case is defined) pending eligibility criterion is fulfilled: same age (in year) and calendar year at cohort entry.

### **6.4.2. Exposure**

Several groups of drug exposure will be considered taking in account the number of months where a drug reimbursement will be observed (including periods during which patients may have received the drug under a pre-marketing authorization, i.e. "ATU" status). It should be noted that before 2012, the “ATU” status is poorly identified in the SNDS database. But considering that our cohort selection encompasses no ADT in 2011-2012 and that the use of abiraterone or enzalutamide under “ATU” imposes necessarily an ADT use, we will exclude indirectly patients under “ATU” status over the 2011-2012 period. The end of exposure will be defined as the theoretical date at which a subsequent reimbursement should have been done.

Drug exposure will be a continuous variable.

Before and after the introduction of abiraterone and enzalutamide, drug switches toward hormonal therapies or chemotherapy will be identified.

The date of secondary primary malignancy as identified in the SNDS is assuredly not the date of cancer occurrence, not even the date of clinical suspicion (because there is no available diagnosis, nor symptoms related to medical visit). A lag-time will be introduced: the drug exposure will not be taken in account in the 6 months before the SPC diagnosis.

## 6.5 Potential confounding variables and other risk factors

External beam radiotherapy for a localized prostate cancer has been found at an increased risk of developing a second primary bladder cancer compared with radical prostatectomy^30^. Of note, in this study, there was a lack of information regarding the dose of radiotherapy and furthermore, patients were treated before 2009 and improvements in EBRT technique have occurred since then. A systematic review and meta-analysis of observational studies comparing patients exposed or unexposed to RT demonstrate an increased risk of developing second cancers for bladder (OR: 1.39), colorectal (OR: 1.68), and rectum (OR: 1.62), with similar risks over lag times of 5 and 10 years^2^. Absolute risks over 10 years are small (1–4%). Biochemical progression after treatment of a localized prostate cancer may occur within 2 to 3 years, thus even a lengthy look-back period could miss initial radiotherapy.

There is strong evidence that obesity, smoking and hyperinsulinemia may increase the risk of advanced disease^31^. Several studies showed that higher cholesterol levels are associated with increased risk of advanced prostate cancer. There is a consistent inverse association between statins use and advanced disease^32^; the risk of diseases-specific mortality under metformin use have discussed, with data suggesting a lack of association^31^. European prostate cancer guidelines recommended to consider treatment strategies to reduce the risk of diabetes and cardiovascular disease^1^.

There is no strong evidence supporting that the severity of the disease is related to the risk of secondary primary malignancy. We will try to catch early treatment of prostate cancer (prostatectomy, radiotherapy) to guess diagnosis context.

As regards co-morbidities and co-medications that could have influenced the choice between enzalutamide and abiraterone, small imbalances were described^3^ for hypertension, chronic renal insufficiency, diabetes, atrial fibrillation, heart disease, ischemic stroke, atherosclerosis and peripheral embolism, carotid and peripheral angioplasty, ischemic heart disease and/or coronary revascularisation, alcohol use disorders, use of anticoagulants, antiplatelet inhibitors, drugs affecting bone structure and mineralisation, drug preventing tumour bone complications. They will identify through the same coding system as for exposure and outcome.

Overall, we assume that comorbidities and associated treatments are not confounders because they do not influence drug choice and are not related to SPC risk.

## 6.6. Data analysis

***6.6.1. Descriptive analysis*.**

First, in the cohort study, we will describe the number of patients by year of inclusion, the mean and median age at the time of inclusion. The description of the drug used will be performed (as proxy of the hormone sensitive and mCRPC status) as well as the potential drug switch. Presence of metastases will be identified using an algorithm identification^33^.

We will estimate the crude incidence of SPC among prostate cancer survivors newly exposed to either androgen deprivation therapy alone, ADT + abiraterone or ADT + enzalutamide.

The date of SPC identified in the SNDS is potentially later than in reality. But this misclassification bias is thought to be non-differential when comparing patients exposed to enzalutamide or abiraterone or ADT, under the assumption that medical follow-up is more or less comparable. A description of the number of medical doctor (including specialist) consultation as well as of the reimbursement of cancer screening measures (including biological and imaging) will be performed.

***6.6.2. Primary Analysis*.**

The nested case-control sample will be analysed using conditional logistic regression with the available procedure in SAS software, with adjustment on variables previously described to estimate the odds ratio of SPC in patients who used ‘ADT alone’ (reference group), ‘ADT + abiraterone” or ‘ADT + enzalutamide’. Patients with drug switches will not be considered in this analyse (see sensitivity analyses section).

We will express the protracted drug exposure (abiraterone or enzalutamide) as given continuously in time and will implement latency models exploring a simple step function to get a general idea of the shape of the latency curve, then favouring flexible and interpretable model such as linear or bilinear models^26^.

***6.6.3* *Sensitivity analyses***.

First, to test the impact of the inaccuracy of the date of SPC, we will consider a lag-time of 9 and 12 months where the drug exposure will not be considered in the 9 then 12 months before the SPC diagnosis.

Second, patients for whom a drug change was identified will be considered and included step-by-step to see the effect of each type of drug change (abiraterone to enzalutamide and vice versa, abiraterone/enzalutamide to chemotherapy, etc.) on the risk of SPC.

***6.6.4. Statistical procedures***

Descriptive analyses will use summary statistics for continuous and categorical data. Categories will be combined or results suppressed to avoid reporting any cell counts less than 11, as required by the CNAM Data Use Agreement. This step will describe the cohort according to drug exposure at enrolment (first observed delivery). This step will also describe cases (first secondary primary malignancy) and their age-matched controls.

## 6.7. Study size

Based on the assumption that the odds ratio of enzalutamide versus abiraterone or ADT alone is 1.15, a sample size of 10,000 cases of SPC is required, considering 4 matched controls per case, an exposure proportions being .85, .10 and .05 in controls for ADT, abiraterone, and enzalutamide, respectively, for approximately 80% power (not taking in account matching) and a significance level of 0.025 using a 1-sided test, which is equivalent to 0.05 using a 2-sided test. Only 2-sided p-values will be presented.

As a reminder, around the world, incidence rate of SPC ranged from 13 to 81 per 1000 person-years for metastatic hormone sensitive prostate cancer^20,21^ and from 59 to 116 for per 1000 person-years for mCRPC^21–23^.

Simulation of expected number of cases over 5 years are as follows:

| Number of patients enrolled per year | Attrition rate | Incidence rate of SPC | Number of cases over 5 years |
| --- | --- | --- | --- |
| 15,000 | 10% | 5% | 9,858 |
|  | 5% | 5% | 10,527 |
| 10,000 | 10% | 8% | 10,515 |
|  | 5% | 8% | 11,229 |

In a previous study based on SNDS, we identified 25,500 adult men with prostate cancer who initiated LHRH agonists or antagonist, between 1^st^ July, 2010, and the 31^st^ December, 2011; thus, around 17,000 over one year^6^. In another previous study based on SNDS, over the 2014-2017 period, 10,000 new users of second-generation androgen receptor antagonists (enzalutamide or abiraterone) were identified: 2/3 were prescribed abiraterone, and 1/3 enzalutamide thus, around 2,500 over one year^3^. Hence, we could reasonably expect the following exposure proportions of .85, .10 and .05 in controls for LHRH agonists or antagonists, abiraterone, and enzalutamide, respectively.

**proc** **power**;

twosamplefreq test=fisher alpha=**0.025** sides=**1** groupweights= (**4** **1**)

power=**.8** or = **1.1** **1.15** **1.2** refp = **0.05**

ntotal =**.**;

**run**;

| **Computed N Total** | | | |
| --- | --- | --- | --- |
| **Index** | **Odds Ratio** | **Actual Power** | **N Total** |
| **1** | 1.10 | 0.800 | 110220 |
| **2** | 1.15 | 0.800 | 50515 |
| **3** | 1.20 | 0.800 | 29270 |

### **6.8. Statistical software(s)**

We will use the SAS software hosted by the CNAM gateway.

## 7. Quality control

Team members have a certification delivered by CNAM and will be authorized by ANSM and CNAM to get access to a specific project space, and to extract from SNDS database the base cohort. The number of patients extracted (compared to the expected number), and the stability of reimbursement frequencies over time will be used to check for data completeness. Algorithms to identify endpoints and main exposure will be implanted by two persons and only consistent results will allow to go further. Storage of records and archiving of the statistical programming will be part of CNAM responsibility.

**8. Protection of human subjects**

The study will use secondary data collected from an existing database (SNDS) and informed consent is not required.

The protocol will be validated by the DRUG SAFE® team and submitted to EPIPHARE Scientific Committee.

All data to be used in this study come from existing anonymized records (SNDS). Only aggregate data will be exported for statistical reporting.

# 9. Management and reporting of adverse events/adverse reactions

Reporting is not required.

# 10. Plans for disseminating and communicating study results

The results of the study will be submitted for publication.

Authorship of any publications resulting from this study will be determined on the basis of the International Committee of Medical Journal Editors (ICJME) Recommendations for the Conduct, Reporting, Editing, and Publication of Scholarly Work in Medical Journals.

All results (Scientific Report) based on this study must be submitted to EPIPHARE for review.

# 11. References

1. Cornford P, van den Bergh RCN, Briers E, et al. EAU-EANM-ESTRO-ESUR-SIOG Guidelines on Prostate Cancer. Part II-2020 Update: Treatment of Relapsing and Metastatic Prostate Cancer. *Eur Urol*. 2021;79(2):263-282. doi:10.1016/j.eururo.2020.09.046

2. Wallis CJD, Mahar AL, Choo R, et al. Second malignancies after radiotherapy for prostate cancer: systematic review and meta-analysis. *BMJ*. 2016;352:i851. doi:10.1136/bmj.i851

3. Scailteux L-M, Campillo-Gimenez B, Kerbrat S, et al. Overall survival among chemotherapy-naïve castration-resistant prostate cancer patients under abiraterone versus enzalutamide: a direct comparison based on a 2014-2018 French population study (the SPEAR cohort). *Am J Epidemiol*. 2021;190(3). doi:10.1093/aje/kwaa190

4. Fizazi K, Tran N, Fein L, et al. Abiraterone plus Prednisone in Metastatic, Castration-Sensitive Prostate Cancer. *N Engl J Med*. 2017;377(4):352-360.

5. Rozet F, Hennequin C, Beauval J-B, et al. [CCAFU french national guidelines 2016-2018 on prostate cancer]. *Prog Urol*. 2016;27 Suppl 1:S95-S143.

6. Scailteux L-M, Vincendeau S, Balusson F, et al. Androgen deprivation therapy and cardiovascular risk: No meaningful difference between GnRH antagonist and agonists-a nationwide population-based cohort study based on 2010-2013 French Health Insurance data. *Eur J Cancer*. 2017;77:99-108.

7. James ND, Spears MR, Clarke NW, et al. Survival with Newly Diagnosed Metastatic Prostate Cancer in the “Docetaxel Era”: Data from 917 Patients in the Control Arm of the STAMPEDE Trial (MRC PR08, CRUK/06/019). *Eur Urol*. 2015;67(6):1028-1038. doi:10.1016/j.eururo.2014.09.032

8. Vellky JE, Ricke WA. Development and prevalence of castration-resistant prostate cancer subtypes. *Neoplasia*. 2020;22(11):566-575. doi:10.1016/j.neo.2020.09.002

9. Shore ND, Drake CG, Lin DW, et al. Optimizing the management of castration-resistant prostate cancer patients: A practical guide for clinicians. *The Prostate*. 2020;80(14):1159-1176. doi:10.1002/pros.24053

10. Beckmann K, Garmo H, Franck Lissbrant I, Stattin P. The Value of Real-World Data in Understanding Prostate Cancer Risk and Improving Clinical Care: Examples from Swedish Registries. *Cancers (Basel)*. 2021;13(4):875. doi:10.3390/cancers13040875

11. Xtandi-Risk-management-plan-summary (last update May 12th, 2021).pdf. Accessed August 5, 2021. https://www.ema.europa.eu/en/documents/rmp-summary/xtandi-epar-risk-management-plan-summary_en.pdf

12. Scailteux L-M, Despas F, Balusson F, et al. Hospitalization for adverse events under abiraterone or enzalutamide exposure in real-world setting: A French population-based study on prostate cancer patients. *Br J Clin Pharmacol*. Published online July 5, 2021. doi:10.1111/bcp.14972

13. Zytiga®-assessment-report-variation_Dec2017.pdf. Accessed July 22, 2020. https://www.ema.europa.eu/en/documents/variation-report/zytiga-h-c-2321-ii-0047-epar-assessment-report-variation_en.pdf

14. xtandi-epar-assessment-report-variation_(February 2021).pdf. Accessed August 5, 2021. https://www.ema.europa.eu/en/documents/variation-report/xtandi-h-c-2639-ii-0047-g-epar-assessment-report-variation_en.pdf

15. zytiga-epar-product-information_December 2020.pdf. Accessed August 11, 2021. https://www.ema.europa.eu/en/documents/product-information/zytiga-epar-product-information_en.pdf

16. zytiga-epar-public-assessment-report (July 2011).pdf. Accessed August 5, 2021. https://www.ema.europa.eu/en/documents/assessment-report/zytiga-epar-public-assessment-report_en.pdf

17. zytiga-epar-assessment-report-variation (October 2017).pdf. Accessed August 5, 2021. https://www.ema.europa.eu/en/documents/variation-report/zytiga-h-c-2321-ii-0047-epar-assessment-report-variation_en.pdf

18. Sternberg CN, Fizazi K, Saad F, et al. Enzalutamide and Survival in Nonmetastatic, Castration-Resistant Prostate Cancer. *New England Journal of Medicine*. Published online May 29, 2020. doi:10.1056/NEJMoa2003892

19. xtandi-epar-product-information_June 2021.pdf. Accessed August 11, 2021. https://www.ema.europa.eu/en/documents/product-information/xtandi-epar-product-information_en.pdf

20. Fan C-Y, Huang W-Y, Lin C-S, et al. Risk of second primary malignancies among patients with prostate cancer: A population-based cohort study. *PLoS One*. 2017;12(4):e0175217. doi:10.1371/journal.pone.0175217

21. Mehtälä J, Zong J, Vassilev Z, et al. Overall survival and second primary malignancies in men with metastatic prostate cancer. *PLoS One*. 2020;15(2):e0227552. doi:10.1371/journal.pone.0227552

22. Saltus CW, Vassilev ZP, Zong J, et al. Incidence of Second Primary Malignancies in Patients with Castration-Resistant Prostate Cancer: An Observational Retrospective Cohort Study in the United States. *Prostate Cancer*. 2019;2019:4387415. doi:10.1155/2019/4387415

23. Vassilev ZP, Gabarró MS, Kaye JA, et al. Incidence of second primary malignancies in metastatic castration-resistant prostate cancer: results from observational studies in three countries. *Future Oncol*. 2020;16(25):1889-1901. doi:10.2217/fon-2020-0315

24. Feifel J, Gebauer M, Schumacher M, Beyersmann J. Nested exposure case-control sampling: a sampling scheme to analyze rare time-dependent exposures. *Lifetime Data Anal*. 2020;26(1):21-44. doi:10.1007/s10985-018-9453-4

25. Essebag V, Platt RW, Abrahamowicz M, Pilote L. Comparison of nested case-control and survival analysis methodologies for analysis of time-dependent exposure. *BMC Med Res Methodol*. 2005;5(1):5. doi:10.1186/1471-2288-5-5

26. Langholz B, Thomas D, Xiang A, Stram D. Latency analysis in epidemiologic studies of occupational exposures: application to the Colorado Plateau uranium miners cohort. *Am J Ind Med*. 1999;35(3):246-256. doi:10.1002/(sici)1097-0274(199903)35:3<246::aid-ajim4>3.0.co;2-6

27. Bezin J, Duong M, Lassalle R, et al. The national healthcare system claims databases in France, SNIIRAM and EGB: Powerful tools for pharmacoepidemiology. *Pharmacoepidemiol Drug Saf*. 2017;26(8):954-962.

28. Tuppin P, Rudant J, Constantinou P, et al. Value of a national administrative database to guide public decisions: From the système national d’information interrégimes de l’Assurance Maladie (SNIIRAM) to the système national des données de santé (SNDS) in France. *Rev Epidemiol Sante Publique*. 2017;65 Suppl 4:S149-S167.

29. Ajrouche A, Estellat C, De Rycke Y, Tubach F. Evaluation of algorithms to identify incident cancer cases by using French health administrative databases. *Pharmacoepidemiol Drug Saf*. 2017;26(8):935-944.

30. Moschini M, Zaffuto E, Karakiewicz PI, et al. External Beam Radiotherapy Increases the Risk of Bladder Cancer When Compared with Radical Prostatectomy in Patients Affected by Prostate Cancer: A Population-based Analysis. *Eur Urol*. 2019;75(2):319-328. doi:10.1016/j.eururo.2018.09.034

31. Siltari A, Auvinen A, Murtola TJ. Pharmacoepidemiological Evaluation in Prostate Cancer-Common Pitfalls and How to Avoid Them. *Cancers (Basel)*. 2021;13(4):696. doi:10.3390/cancers13040696

32. Pernar CH, Ebot EM, Wilson KM, Mucci LA. The Epidemiology of Prostate Cancer. *Cold Spring Harb Perspect Med*. 2018;8(12):a030361. doi:10.1101/cshperspect.a030361

33. Thurin NH, Rouyer M, Gross-Goupil M, et al. Epidemiology of metastatic castration-resistant prostate cancer: A first estimate of incidence and prevalence using the French nationwide healthcare database. *Cancer Epidemiol*. 2020;69:101833. doi:10.1016/j.canep.2020.101833

# Annex 1. List of stand-alone documents

| Number | Document reference number | Title |
| --- | --- | --- |
| *1* | Figure-1 | History of prostate cancer |
| *3* | Table 1 | Delivery codes for androgen deprivation therapy |
| 4 | Table 2 | Other cancer than prostate cancer, except for non-melanoma skin cancer |

Figure-1


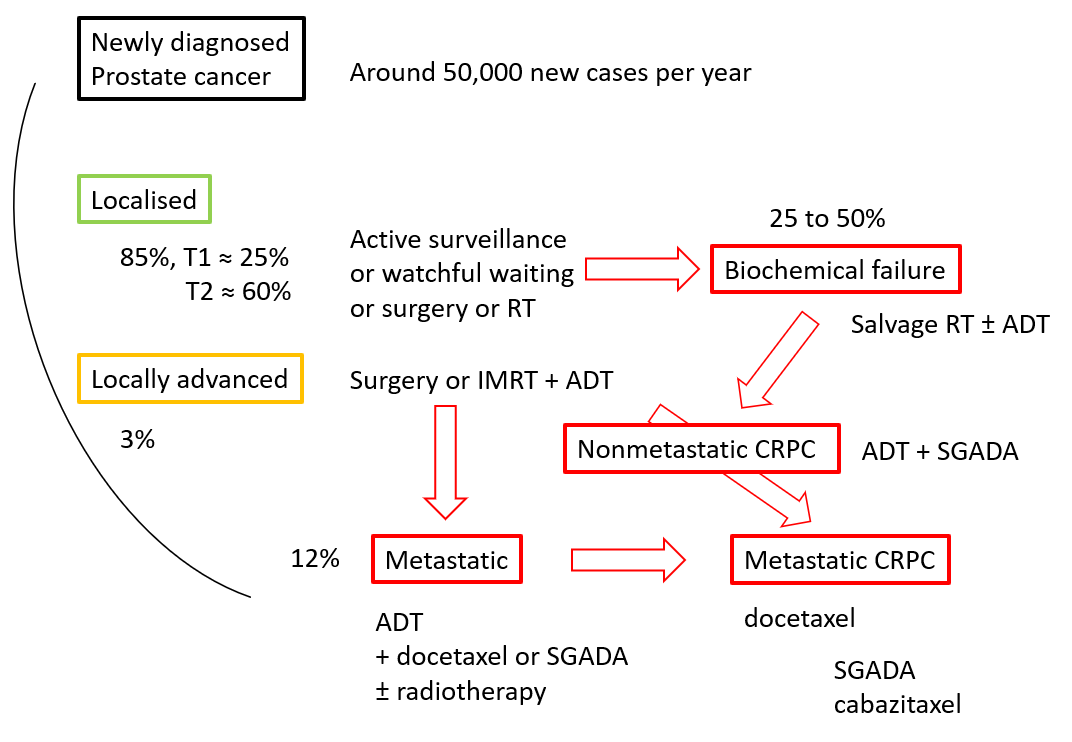


Table 1 Delivery codes for androgen deprivation therapy

| **CIP label** | **CIP13** | **UCD13** | **UCD7** | **ATC** | **DCI** |
| --- | --- | --- | --- | --- | --- |
| **Gonadotropin releasing hormone analogues** | |  |  |  |  |
| ELIGARD 22,5MG INJ SRG+SRG 1 | 3400936690929 | 3400892815619 | 9281561 | L02AE02 | Leuprorelin |
| ELIGARD 45MG INJ SRG+SRG PLAT1 | 3400938263381 | 3400893090879 | 9309087 | L02AE02 |  |
| ELIGARD 7,5MG INJ SRG+SRG 1 | 3400936690868 | 3400892815787 | 9281578 | L02AE02 |  |
| ENANTONE LP 11,25MG INJ SRG 1 | 3400930122129 | 3400894387329 | 9438732 | L02AE02 |  |
| ENANTONE LP 3,75MG INJ F+A.1+D | 3400937575256 | 3400892922638 | 9292263 | L02AE02 |  |
| ENANTONE LP 3,75MG INJ SRG 1 | 3400930122112 | 3400894387497 | 9438749 | L02AE02 |  |
| ENANTONE LP 30MG INJ SRG 1 | 3400938458381 | 3400893167793 | 9316779 | L02AE02 |  |
| LEPTOPROL 5MG IMPL SRG 1 | 3400930020005 | 3400894355380 | 9435538 | L02AE02 |  |
| ZOLADEX 10,8MG IMPL INJ SRG 1 | 3400933963811 | 3400891830606 | 9183060 | L02AE03 | Goserelin |
| ZOLADEX 3,6MG IMPL INJ SRG 1 | 3400932932672 | 3400891309225 | 9130922 | L02AE03 |  |
| DECAPEPTYL 0,1MG F+A.AIPBBAES7 | 3400949003266 | 3400890000307 | 9000030 | L02AE04 | Triptorelin |
| DECAPEPTYL 0,1MG INJ FL+AMP 7 | 3400932850266 | 3400891094190 | 9109419 | L02AE04 |  |
| DECAPEPTYL LP 3MG FL+AMP 1+NEC | 3400933943769 | 3400891769012 | 9176901 | L02AE04 |  |
| DECAPEPTYL LP11,25MG AIPBBA PL | 3400949002634 | 3400894431763 | 9443176 | L02AE04 |  |
| DECAPEPTYL LP11,25MG AIPMWI PL | 3400949003938 | 3400890008358 | 9000835 | L02AE04 |  |
| DECAPEPTYL LP11,25MG AIPPLA PL | 3400949002818 | 3400894462521 | 9446252 | L02AE04 |  |
| DECAPEPTYL LP11,25MG F+A 1+NEC | 3400934125652 | 3400891864083 | 9186408 | L02AE04 |  |
| DECAPEPTYL LP22,5MG FL+A 1+NEC | 3400939890173 | 3400893457177 | 9345717 | L02AE04 |  |
| GONAPEPTYL 3,75MG INJ SRG+SRG1 | 3400935844675 | 3400892765020 | 9276502 | L02AE04 |  |
| SALVACYL LP 11,25MG INJ FL+A.1 | 3400938081459 | 3400893242995 | 9324299 | L02AE04 |  |
| **Anti-androgens** |  |  |  |  |  |
| ANANDRON 150MG CPR BT30 | 3400933264529 | 3400891703306 | 9170330 | L02BB02 | Nilutamide |
| ANANDRON 50MG CPR BT90 | 3400932882724 | 3400891221992 | 9122199 | L02BB02 |  |
| BICALUTAMIDE ACC 50MG CPR BT30 | 3400949467020 | 3400893590331 | 9359033 | L02BB03 | Bicalutamide |
| BICALUTAMIDE ALM 50MG CPR BT30 | 3400938588293 | 3400893199503 | 9319950 | L02BB03 |  |
| BICALUTAMIDE ARL 50MG CPR BT30 | 3400937728263 | 3400892964102 | 9296410 | L02BB03 |  |
| BICALUTAMIDE BGA 50MG CPR BT30 | 3400937851770 | 3400893027196 | 9302719 | L02BB03 |  |
| BICALUTAMIDE CRP 50MG CPR BT30 | 3400930181256 | 3400890008112 | 9000811 | L02BB03 |  |
| BICALUTAMIDE EG 50MG CPR BT30 | 3400937611657 | 3400892913124 | 9291312 | L02BB03 |  |
| BICALUTAMIDE EVO 50MG CPR BT30 | 3400939318516 | 3400893366356 | 9336635 | L02BB03 |  |
| BICALUTAMIDE KBI 50MG CPR BT30 | 3400937577038 | 3400893517352 | 9351735 | L02BB03 |  |
| BICALUTAMIDE MYL 50MG CPR BT30 | 3400938432886 | 3400893169056 | 9316905 | L02BB03 |  |
| BICALUTAMIDE SDZ 50MG CPR BT30 | 3400938520347 | 3400893152546 | 9315254 | L02BB03 |  |
| BICALUTAMIDE TVC 50MG CPR BT30 | 3400938407815 | 3400893152775 | 9315277 | L02BB03 |  |
| BICALUTAMIDE ZEN 50MG CPR BT30 | 3400937938402 | 3400893152836 | 9315283 | L02BB03 |  |
| BICALUTAMIDE ZYD 50MG CPR BT30 | 3400921957754 | 3400893816820 | 9381682 | L02BB03 |  |
| CASODEX 50MG CPR BT30 | 3400933916336 | 3400891764628 | 9176462 | L02BB03 |  |
| XTANDI 40MG CPR BT112 | 3400930117644 | 3400894294665 | 9429466 | L02BB04 | Enzalutamide |
| XTANDI 40MG CPR BT112 | 3400958901911 | 3400894294665 | 9429466 | L02BB04 |  |
| ERLEADA 60MG CPR BT120 | 3400930167267 | 3400894448853 | 9444885 | L02BB05 | Apalutamide |
| NUBEQA 300MG CPR BT112 | 3400930203545 | 3400890007177 | 9000717 | L02BB06 | Darolutamide |
| **Other hormone antagonists and related agents** | |  |  |  |  |
| FIRMAGON 120MG INJ FL+FL 2 | 3400939432748 | 3400893459997 | 9345999 | L02BX02 | Degarelix |
| FIRMAGON 80MG INJ FL+FL 1 | 3400939432687 | 3400893460078 | 9346007 | L02BX02 |  |
| ZYTIGA 500MG CPR BT60 | 3400930076279 | 3400894237136 | 9423713 | L02BX03 | Abiraterone Acetate |
|  |  |  |  |  |  |
|  |  |  |  |  |  |
| **CIP label** | **CIP13** | **UCD13** | **UCD7** | **ATC** | **DCI** |
| **Antiandrogens, plain** |  |  |  |  |  |
| ANDROCUR 50MG CPR BT20 | 3400932351008 | 3400890047906 | 9004790 | G03HA01 | Cyproterone |
| CYPROTERONE ARW 100MG CPR BT60 | 3400938844597 | 3400893238226 | 9323822 | G03HA01 |  |
| CYPROTERONE ARW 50MG CPR BT20 | 3400938844368 | 3400893238394 | 9323839 | G03HA01 |  |
| CYPROTERONE BGA 100MG CPR BT60 | 3400934201684 | 3400892902296 | 9290229 | G03HA01 |  |
| CYPROTERONE BGA 50MG CPR BT20 | 3400934232732 | 3400892575162 | 9257516 | G03HA01 |  |
| CYPROTERONE EG 100MG CPR BT60 | 3400936945814 | 3400892784410 | 9278441 | G03HA01 |  |
| CYPROTERONE EG 50MG CPR BT20 | 3400936269705 | 3400892570600 | 9257060 | G03HA01 |  |
| CYPROTERONE MYL 100MG CPR BT60 | 3400936935297 | 3400892784878 | 9278487 | G03HA01 |  |
| CYPROTERONE MYL 50MG CPR BT20 | 3400934090141 | 3400891844733 | 9184473 | G03HA01 |  |
| CYPROTERONE TVC 100MG CPR BT60 | 3400938206203 | 3400893094433 | 9309443 | G03HA01 |  |
| CYPROTERONE TVC 50MG CPR BT20 | 3400938205251 | 3400893087978 | 9308797 | G03HA01 |  |

Table 2 Other cancer than prostate cancer, except for non-melanoma skin cancer

| Anatomic site | ICD-10 codes |
| --- | --- |
| Lip, oral cavity and pharynx  Digestive organs  Respiratory and intrathoracic organs (non-lung)  Lung  Bone and articular cartilage  Skin §  Mesothelial and soft tissue  Male genital organs §  Urinary tract  Central nervous system including eye  Endocrine glands  Lymphoid tissue  Haematopoietic tissue  Other \|\| | C00-14  C15-26  C30-33, C35-39, C450  C34  C40-41  C43-44  C45-49  C60-63  C64-68  C69-72, C751-753  C73-74, C750, C754-759  C81-90  C91-96  C76-80, C97 |

§ Excludes basal cell carcinoma

|| Cancers of ill-defined, secondary and unspecified sites and cancers of independent multiple primary sites.

# Budget prévisionnel (en euros)

|  | Dépenses directes liées à l'exécution du projet | Dépenses éligibles |
| --- | --- | --- |
| **Dépenses de personnel** |  |  |
| *Responsable coordonnateur* (5% pdt 10 mois, 1 ETP = 120 000€/an) | 5000 | Non éligible |
| *Responsable Scientifique, membre du CS* (temps cumulé 5 mois-homme,  1 ETP = 90 000€/an) | 37500 | Non éligible |
| *Gestionnaire de données niveau ingénieur* (4 mois-homme, 70 000€/an) | 23333 | 23333 |
| *Statisticien* (2 mois-homme, 90 000€/an) | 15000 | 15000 |
| **Dépenses de fonctionnement** | 1500 | 1500 |
| **Dépenses d'équipement** | 2000 | 2000 |
| **Frais de gestion (4%)** | 3373 | 1673 |
| Total | 87706 | **43506** |
